# Supplementary material for: Ecological diversification of sea catfishes is accompanied by genome-wide signatures of positive selection
Source: Nat Commun. 2024 Nov 20;15:10040. doi: 10.1038/s41467-024-54184-3 (PMC11579386; doi:10.1038/s41467-024-54184-3)
Supplement: Supplementary file 1 — Supplementary Information [file 41467_2024_54184_MOESM1_ESM.pdf]

Supplementary Information

Supplementary Materials and Methods
Supplementary Notes 1-8
Figures S1-S21
Supplementary Tables S1-S2
Supplementary References
Supplementary Data S1-S17

Supplementary Materials and Methods

DNA extractions, library preparation and sequencing. We extracted total DNA from 20 mg of muscle or fins using the DNeasy Blood & Tissue Kits from QIAGEN® and quantified the genomic DNA using a Qubit fluorometer (Life Technologies, Inc.).

For the samples sequenced using the exon capture approach, Daicel Arbor Biosciences (Ann Arbor, MI) performed library preparation using an Illumina TruSeq-style library preparation protocol. Target capture probes were designed based on alignments of 1,051 single-copy exons optimized for otophysans (Otophysi baitset[1]). Probes were synthesized with a myBaits custom probe. Samples were sequenced on an Illumina HiSeq 2500 platform at 100bp pair-end.

For the whole-genome short-read ('shotgun') sequencing approach, we sent high-quality DNA samples to four different facilities: Daicel Arbor Biosciences (Ann Arbor, MI), Novogene Corporation Incorporated (Sacramento, CA), GENEWIZ from Azenta Life Sciences (South Plainfield, NJ), and Oklahoma Medical Research Foundation (Oklahoma City, OK). Each facility prepared individual libraries using the Illumina TruSeq Library Construction Kit for insert size 350 bp and sequenced them at 150bp pair-end on an Illumina HiSeq 2500 and Illumina NovaSeq S4 platforms, aiming for ~30X coverage assuming a genome size of 2.04-2.42 Gbp based on data from three ariid species: *Neoarius graeffei* (reference genome), *Ariopsis canteri, and Brustiarius solidus* (survey genomes; see below).

Reference genome sequencing and assembly. A subadult lesser salmon catfish (*Neoarius graeffei*) was collected from Barratta Creek, Australia (-19.568° S, 147.207° E) on August 7, 2020. Flash-frozen samples (blood, skin, fin clips, gill rakers, muscle) preserved in ethanol and RNA later were sent to the Vertebrate Genome Laboratory (VGL) for DNA/RNA extraction, sequencing, and assembly. High molecular weight DNA, isolated from blood using the Bionano Prep SP Frozen Blood DNA Isolation protocol v2 (Bionano Genomics), was quantified (Qubit 3 fluorometer) and assessed for fragment length (Agilent Fragment Analyzer).

VGL generated a reference genome using third-generation sequencing technologies and an iterative assembly pipeline v2.0[2]. DNA (6 µg) was sheared (Megaruptor 3) before PacBio library preparation (SMRTbell Express Template Prep Kit 2.0) and size selection (>10 kb). The library was sequenced on a Sequel IIe instrument. Bionano optical maps were generated from 750 ng DNA using direct labeling (DLE1) and the Bionano Prep DLS protocol. Hi-C data (67x) was produced using the Arima-HiC 2.0 kit (Arima Genomics). For Iso-Seq data, total RNA was extracted (QIAGEN RNAeasy Protect kit) and a PacBio Iso-Seq Express 2.0 library was prepared and sequenced on a Sequel IIe instrument.
The assembly process included genome profile analysis, phased assembly of HiFi long reads (hifiasm[3]), contig removal (purge_dups pipeline), and scaffolding using Bionano and Hi-C data. Quality control involved QUAST[4], BUSCO[5,6], Merqury[7], and Pretext[8]. For Iso-Seq, the pipeline masked genomic sequences, aligned transcripts, proteins, and RNA-Seq reads to the genome, predicted gene models, and assigned names and accessions. The final assembly, annotations, and associated data are available under NCBI accession GCF_027579695.1.

Survey genomes. We used Novogene Corporation Incorporated genomic services and solutions to survey the genomes of two ariid species: *Ariopsis canteri* and *Brustiarius solidus*. They extracted DNA, prepared a 350bp insert DNA library using the Illumina TruSeq Library Construction Kit, and sequenced it using an Illumina NovaSeq platform 150 pair-end. They also performed bioinformatic analysis which included data quality control, analysis of k-mer frequency, preliminary genome assembly with SOAPdenovo[9], and inspection for contamination. The survey results for *Ariopsis canteri* show a genome size of 2046.67 Mbp, heterozygous rate of 0.28%, and repeat content of 69.95%. For *Brustiarius solidus*, the survey results show a genome size of 2393.73 Mbp, a heterozygous rate of 0.29%, and a repeat content of 74.19%.

Assembly of short-read genomes. Illumina raw reads were quality checked with FastQC v0.11.5[10] and filtered using Trimmomatic v0.38[11] to remove low-quality bases and adapters. We assembled the resulting trimmed sequences with SPAdes v3.13.1[12] and removed contaminants using NCBI's Foreign contamination Screen (FCS) tool (https://github.com/ncbi/fcs). To improve the quality of the assemblies, we used RagTag v2.1.0[13] to scaffold the SPAdes preassemblies as well as the survey genome assemblies. RagTag used the lesser salmon catfish reference genome to scaffold the assemblies, arranging and aligning the sequences, and adding gaps to connect them, without any modification to the input query sequence.

Assembly of exon capture data and mining of exons from the genomes. We assembled the raw reads for 18 species into loci using the FishLifeExonCapture pipeline (https://github.com/lilychughes/FishLifeExonCapture). Briefly, we first trimmed the raw reads for low-quality base calls and adapter contamination with Trimmomatic v0.38[11]. Next, we mapped the trimmed reads against the representative Otophysi 1,051 sequences using BWA v0.7.17[14], and removed PCR duplicates with SAMtools v1.9[15]. We generated initial assemblies with Velvet v1.2.07[16], and used the longest assembled contig as a reference for aTRAM v2.2[17]. We ran aTRAM for a maximum of five iterations and removed redundant contigs with CD-HIT v4.8.1[18] with a threshold of 98% similarity. To find reading frames, we used the coding2genome algorithm in Exonerate v2.4.0[19], to align the assembled contig to an Otophysi reference sequence previously verified by visual inspection. Finally, we aligned the exons using the reading-frame-aware aligner MACSE v2.03[20].

For each of the 1,051 target sequences from the Otophysi baitset, we parameterized an HMM profile and executed a nHMMER search on each genome, using default settings in HMMER v3.2.1[21]. Hits were obtained for each marker for each of the species and extracted from genomes with custom Python scripts (https://github.com/lilychughes/FishLifeExonHarvesting). After performing standard procedures for sequence quality control and assembly, we aligned exons using MACSE v2.03. All aligned sequences were checked visually in Geneious Prime® v2022.1[22]. We concatenated individual exon alignments into a supermatrix consisting of 1,039 genes and 119 taxa (255,570 bp).

Legacy markers. To build phylogenetic trees, we expanded our taxonomic sampling from exon capture and WGS by adding 36 more species. We assembled a supermatrix by combining the 1,051 exon markers with multi-locus sequence data from previous studies[23–25]. This supermatrix included three nuclear legacy markers (MYH6, RAG1, RAG2) as well as four mitochondrial markers (12S, 16S, ATPase 6/8, CYTB), all generated via PCR and Sanger sequencing[23–25].

Phylogenomic analyses of exon markers, divergence time estimation and phylogenetic uncertainty in downstream analyses. To account for phylogenetic and divergence time uncertainty in downstream phylogenetic and comparative genomic analyses, we employed two main strategies for assembling subsets from the expanded matrix. These subsets overlapped only in legacy markers, thereby maintaining the same set of species across subsets. The first strategy involved creating 12 largely independent subsets, each consisting of 93 loci, while the second strategy consisted of four independent subsets, each with 265 loci (Supplementary Data S2). The analyses revealed that using more, albeit smaller, gene subsets of 93 loci resulted in higher phylogenetic disparity (error), as estimated via multidimensional scaling (MDS) of a tree space plot using the R package phytools v1.2-0[26] (Figure S1). Therefore, downstream analyses used

trees inferred from four subsets of 265 markers, as well as the complete dataset (see main text). Resulting trees capture the uncertainty in divergence times and phylogenetic relationships by incorporating different underlying data, which differs from the common practice of using "pseudo-replicated" trees derived from a Bayesian posterior distribution estimated from a single data set, often limited to a few genes (see also [27–29]). Through the use of a sample of topologies spanning the tree space using both concatenation and coalescent approaches, our analyses additionally account for gene tree-species tree discordances that arise from incomplete lineage sorting, which can produce false positives in estimates of positive selection[30].

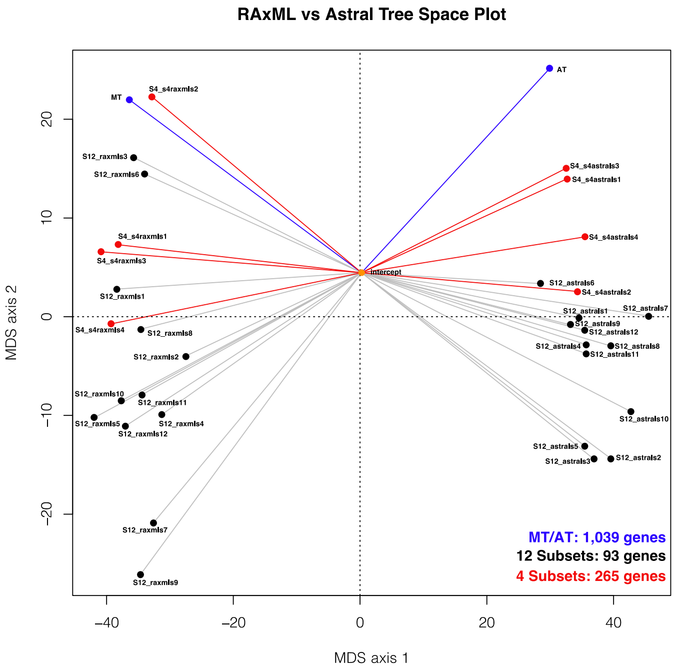

Figure S1. Tree spaces for the trees estimated in this study including a comparison for the two subset approaches. MT: 'master tree', AT: alternative ASTRAL-III tree based on the full dataset. The orange dot represents the average (centroid) tree in tree space. Tree space plot depicting in black the 12 subsets scheme and the four subsets scheme approaches in red. The 12 subsets scheme containing fewer markers per subset shows greater phylogenetic disparity (error) compared to the four-subsets scheme.

Prior to phylogenetic analyses, the best-fitting partitioning scheme was determined for complete datasets and subsets using PartitionFinder v2.1.1[31]. In each case, maximum-likelihood (ML) trees were estimated in RAxML-NG v0.9.0[32] using the best-fit partition selected via the Bayesian Information Criterion (BIC), the GTR model, and a heuristic tree search strategy. The number of bootstrap replicates was determined automatically via the autoMRE function in RAxML-NG. Next, we estimated individual gene trees in RAxML-NG using by-codon partitions based on sequence alignments from all individual loci. Finally, gene trees were used as input for coalescent-based analyses in ASTRAL-III v5.7.1[33]. For tree topology comparison purposes, we also conducted phylogenetic analyses using two alternative approaches. First, protein sequences based on the 3,519 genes identified BUSCO for each species were

used as input OrthoFinder v2.3.3[34] to produce a species tree using Species Tree from All Genes (STAG[35]), which reconciles paralogues, co-orthologues, or other gene duplication events. Second, the OrthoFinder runs also identified 432 single copy orthologue sequences, which we used as input for concatenation-based ML and coalescent-based analyses using both RAxML-NG and ASTRAL-III, respectively.

Finally, to time-calibrate the resulting trees obtained with RAxML-NG and ASTRAL-III, we used the MCMCTree package, as implemented in the program PAML v4.9h[36], which can handle genome-scale datasets in a Bayesian framework[37]. These analyses used only two partitions (1st+2nd and 3rd codon positions) and the 14 calibration points from two previous studies[25,38] (Supplementary Table S1; Figure S2 for additional details).

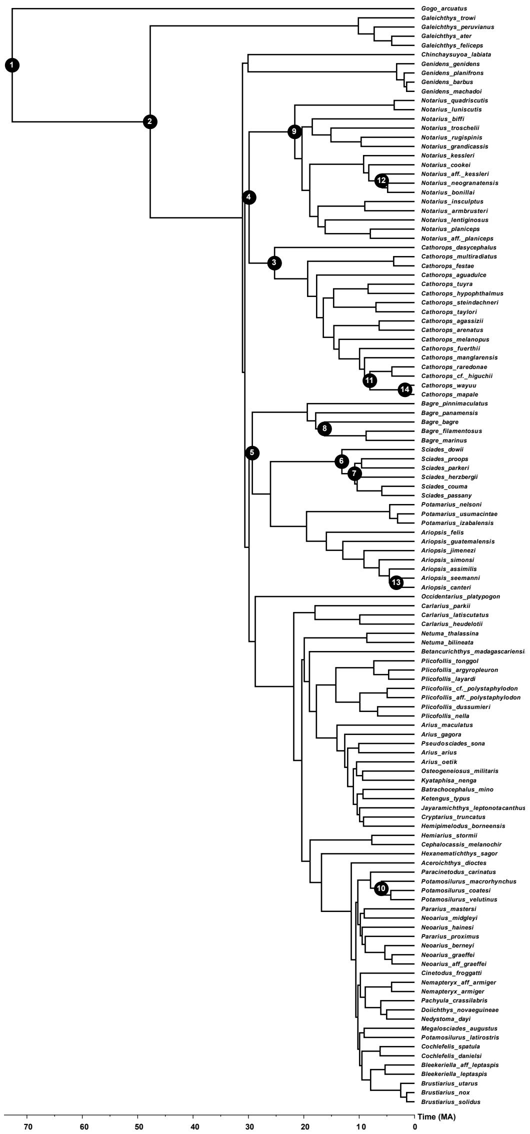

Figure S2. Calibration points. Ariid 'master tree' (full dataset, RAxML) showing the fourteen calibration points used as priors in MCMCTree.

Supplementary Table S1. Priors and calibration points used for divergence time estimations in MCMCTree based on previous studies.

Calibration No.	Node	MRCA	Lower bound (Ma)	Upper bound (Ma)	Distribution	MCMCTree parameters	Calibration type	Justification for calibration	Reference for calibration	Notes
1	Ariidae + Anchariidae	*Gogo* + *Galeichthys*	70.5	80.7	Gamma	G(500,684.9315)	Secondary calibration	Secondary calibration based on Betancur-R. et al. (2012); the primary calibration from that study used ariid fossils of Late Campanian–Early Maastrichtian age (70 Ma)	Betancur-R et al. (2012)[25]	*
2	Ariidae	*Galeichthys* + *Cathorops*	35.6	59.2	Gamma	G(500,1063.83)	Secondary calibration	Secondary calibration based on Betancur-R. et al. (2012)	Betancur-R et al. (2012)[25]	*
3	*Cathorops*	*C. dasycephalus*, *C. mapale*	20.4	-	Uniform	L(0.204,0.1,1,1e-300)	Fossil skull	*Cathorops goeldii* (Pirabas form.; 20.4-23.03 Ma)	Betancur-R et al. (2012)[25]; Stange et al. (2018)[38]	
4	*Notarius*	*Notarius* + sister genus	20.4	-	Uniform	L(0.204,0.1,1,1e-300)	Fossil otolith	*Notarius* sp. (Pirabas form.; 20.4-23.03 Ma)	Stange et al. (2018)[38]	**
5	*Bagre*	*Bagre* + sister genus	20.4	-	Uniform	L(0.204,0.1,1,1e-300)	Fossil otolith	*Bagre protocaribbeanus* (Pirabas form.; 20.4-23.03 Ma)	Stange et al. (2018)[38]	***
6	*Sciades*	*S. dowii* + *S. parkeri*	5.3	20.4	Uniform	-	Fossil skull	*S. dowii* (Urumaco Formation; 5.3-11.63 Ma)	Stange et al. (2018)[38]	****
7	*Sciades* (Caribbean clade)	*S. proops* + *S. herzbegii*	5.3	20.4	Uniform	B(0.053,0.24,1e-300,0.05)	Fossil skull	*S. herzbegii* (Urumaco Formation; 5.3-11.63 Ma)	Stange et al. (2018)[38]	****
8	Bagre (Caribbean clade)	*Bagre marinus/filamentosus* + *B. bagre*	5.3	20.4	Uniform	B(0.053,0.24,1e-300,0.05)	Fossil skull	*B.* aff. *marinus* (Urumaco Formation; 5.3-11.63 Ma)	Stange et al. (2018)[38]	****
9	*Notarius quadriscutis/luniscutis*	*Notarius quadriscutis/luniscutis* + remaining *Notarius*	5.3	20.4	Uniform	B(0.053,0.24,1e-300,0.05)	Fossil skull	*Notarius quadriscutis* (Urumaco Formation; 5.3-11.63 Ma)	Stange et al. (2018)[38]	****, *****
10	*Potamosilurus*	*P. macrorhynchus* + *P. velutinus*	4	8	Uniform	B(0.04,0.08,1e-300,1e-300)	Geological	Uplift of New Guinea Central Mountain range	Betancur-R et al. (2012)[25]	
11	Trans-isthmian *Cathorops*	*Cathorops* (Caribbean) + *Cathorops* (Pacific)	2.8	-	Uniform	L(0.028,0.1,1,1e-300)	Geological	Final rising of Panama isthmus	Betancur-R. et al. (2012)[25]; Rincon-Sandoval et al. (2020)[27]	******, *******
12	Trans-isthmian *Notarius*	*Notarius* aff. *kessleri* + *N. neogranatensis*	2.8	-	Uniform	L(0.028,0.1,1,1e-300)	Geological	Final rising of Panama isthmus	Betancur-R. et al. (2012)[25]; Rincon-Sandoval et al. (2020)[27]	******, *******
13	Trans-isthmian *Ariopsis*	*Ariopsis seemanni* + *Ariopsis canteri*	2.8	-	Uniform	L(0.028,0.1,1,1e-300)	Geological	Final rising of Panama isthmus	Betancur-R. et al. (2012)[25]; Rincon-Sandoval et al. (2020)[27]	******
14	Sierra Nevada S. Marta Massif -- *C. mapale/C. wayuu*	*Cathorops mapale* + *C. wayuu*	0.8	1.2	Uniform	B(0.008,0.012,1e-300,1e-300)	Geological	Northward displacement of Santa Marta Massif	Betancur-R. et al. (2010, 2012)[24,25]	

* These are secondary calibrations. The estimated origin of siluriforms according to molecular dating analyses (e.g, ~150 mya; Lundberg et al. 2007[39]) is much older than predicted by the oldest fossils dated from the Late Campanian-Early Maastrichtian (73–68 mya), suggesting a taphonomic bias in the catfish fossil record. Thus, we constrained two basal nodes (ariids and arioids (= ariids + anchariids)) using secondary calibrations from Betancur-R. et al. (2012)[25]. That study used an extensive selection of bonyfish outgroups to avoid estimating overly old root ages for both ariids and arioids. Because we did not include those outgroups in our alignments, we used instead secondary calibrations based on a gamma distribution drawn around the mean and the 95% HPD age estimates obtained previously for both nodes: 1- mean 73.0 Ma, 95% HPD 70.5-80.7 Ma; 2- mean 46.9 Ma, 95% HPD 35.6-59.2 Ma.

** The sister genus of the clade *Notarius* is unstable; the MRCA was adjusted on a tree-by-tree basis.

*** The sister genus of *Bagre* is unstable; the MRCA was adjusted on a tree-by-tree basis.

**** The upper bound is set to 20.4 based on the fossils from the Pirabas formation (calibrations No. 2-4). This calibration is intended to be placed in the stem lineage of *S. dowii*. However, because MCMCTree does not allow the implementation of stem calibrations, this is effectively redundant with calibration 6 and therefore is not used here.

***** The sister clade of *Notarius quadriscutis/luniscutis* is unstable; the MRCA was adjusted on a tree-by-tree basis.

****** See also Stange et al. (2018)[38] for a close age estimate for the MRCA for the *Cathorops* transisthmian pair (2.58 Ma) without the use of a geological calibration. The minimum age of 2.8 Ma is chosen based on the last undisputed age of the rising of the isthmus; an older upper bound is assigned due to the controversial dates of this event based on recent studies. See comments in Rincon-Sandoval et al. (2020)[27].

*******The transisthmian clades in *Cathorops* and *Notarius* are unstable; the MRCA was adjusted in each case on a tree-by-tree basis.

Reconstruction of ancestral habitats. For the habitat occupancy dataset (Supplementary Data S4), we assigned species into major habitat categories (i.e., marine, euryhaline, freshwater, benthic, pelagic planktivore) by aggregating information from a wide range of sources from the primary literature[25,40,41]. We conducted three independent analyses for each dataset: 1) transitions between marine and freshwater habitats, where euryhaline species were coded with ambiguity (see below); 2) transitions between stenohalinity to euryhalinity, where marine and freshwater species were coded as stenohaline and those present in both habitats as euryhaline; and 3) transitions along the benthic-pelagic axis in the water column, where pelagic planktivore species were codified based on high gill raker counts as a proxy[25,42–44] (see below). To address instances where habitat occupancy is polymorphic (e.g., euryhaline species; Supplementary Data S3), we conducted ancestral character reconstructions that accounted for tip-state ambiguity through stochastic character mapping analyses (SIMMAP[45]) using the R package phytools v1.2-0 (see http://blog.phytools.org/2023/05/fitting-discrete-character-evolution.html). We then assessed the suitability of three models of discrete state evolution for binary states, including equal (ER) or symmetric rates, all rates different (ARD) or asymmetric rates, and symmetric (SYM) transition model, and used the best fit model to determine the distribution of habitat transition events over the course of ariid evolution using SIMMAP. Across the three ecological axes, transitions were counted when a nodal pie is >50% of one color and one of its descendant branches is >50% of the other color. The range of transitions for HyPhy analyses is often less than the total number of transitions counted due to fewer species with genomic data available. For HyPhy analyses based on marine-to-freshwater transitions, euryhaline tips with polymorphic habitat probabilities were either coded based on the most likely tip state inferred with SIMMAP or excluded from the analyses (see episodic diversifying selection section below for details).

For water column transitions, identifying pelagic species in Ariidae poses a challenge, as there is limited information available on habitat affiliation. Instead, we differentiate between benthic and pelagic planktivore species using gill raker counts as a proxy, a trait that correlates with diet composition and prey size. Pelagic planktivore species with a high gill raker count are predicted to have better feeding efficiency on zooplankton, whereas those with low gill raker counts tend to forage on benthic habitats[46–51]. To assign ariid species as benthic or pelagic planktivores, we used gill raker counts for either multiple individuals examined previously[4] or based on the median number of rakers from the range given in the literature[42,44,52–54]. A total of six pelagic planktivores species identified here have 28–59 rakers on the first gill arch, whereas the remaining 113 species coded as benthic have 5–25 rakers. While species classified as pelagic planktivores were confirmed to feed on zooplankton through dietary information (Supplementary Table S2), it is possible that species with low gill raker counts may also be pelagic, leading to a potential underestimation of benthic-to-pelagic transitions in Ariidae. Nevertheless, by

focusing on pelagic planktivores, we were able to examine the evolutionary trajectories of a subset of species with a more restricted trophic ecology along the benthic-pelagic axis.

Supplementary Table S2. Gill raker counts on first gill arch and dietary information for pelagic planktivore species.

Species	Gill rakers (median counts)	Diet information
Brustiarius nox	59	Feeds on large crustaceans such as *Macrobrachium*, large insects and nymphs, fishes (usually *Ophieleotris aporos*), leeches, earthworms, plants and detritus, zooplankton and planktivorous crustaceans, terrestrial insects.[44,55]
Cathorops hypophthalmus	39	Diet: bony fishes, mobile benthic crustacea shrimps/crabs, mobile benthic worms, mobile benthic gastropods/bivalves. The gracile dentition of *C. hypophtalmus* is presumably related to a planktivore diet, although this has not, been determined by stomach content analysis. "With twice the number of gill-rakers than those of the other TEP ariid catfish, it probably feeds mostly on plankton.[40,54]
Doiichthys novaeguineae	48	Consume fine particles, such as suspended plant material and zooplankton, small insect larvae and fine detritus.[42]
Genidens planifrons	28	Filter feeder; eats plankton including *Mysidopsis* shrimps and *Micropogonias furnieri* eggs.
Nedystoma dayi	36	Feeds mainly on aquatic insect larvae; filter feeder.[52,55,56]
Neoarius hainesi	35	Stomach content: aquatic insect larvae (Chironomidae), gastropod mollusks, crabs. Consume small aquatic invertebrates, and different food items derived from the floodplain.[53,57]

Identification of candidate and non-candidate genes for PhyloG2P analyses. We compiled a dataset of 2,310 genes, including 249 candidate genes obtained from the literature (Figure S3), 96 single-copy genes from the Actinopterygii OrthoDB, and 1,965 single-copy orthologs shared among seven Siluriformes species with reference genomes available (see below). The candidate genes dataset comprised genes related to functional categories linked to habitat transitions (see details in main text and below). We identified a set of high-confidence non-candidate orthologs based on genes obtained with the Actinopterygii OrthoDB in BUSCO for 63 ariid species that had high-quality genome assemblies. The outgroup *Gogo arcuatus* and the genome assemblies of *Nemapteryx aff. armiger* and *Osteogeneiosus militaris* were excluded from this analysis due to their low completeness and high fragmentation. Initially, we identified a total of 3,466 single-copy genes in BUSCO. These genes were further parsed through OrthoFinder, resulting in 432 single-copy orthogroups with orthologs in all selected species, although not every species was present in each orthogroup. Subsequently, 109 non-candidate BUSCO genes were retained, which were present in at least 70% of the species, had at least 70% sequence similarity, and had a sequence length greater than 200 bp. All alignments and gene trees were visually inspected in Geneious Prime® v2022.1 to identify additional instances of paralogy, resulting in the retention of 96 genes for downstream analysis. In addition to the BUSCO genes, the largest set of non-candidate 1,965 single-copy

orthologs was derived from a mined dataset comprising 2,453 genes out of a pool of 3,208 genes, sampled using the same set of filters (>70% species, >70% similarity, >200 bp). These genes belong to a set of single copy orthologs shared among the complete annotated set of proteins from seven Siluriformes species in addition to our reference genome, *Neoarius graeffei*. These species are *Tachysurus fulvidraco* (GCF_022655615.1), *Silurus meridionalis* (GCF_014805685.1), *Pangasianodon hypophthalmus* (GCF_027358585.1), *Clarias gariepinus* (GCF_024256425.1), *Ictalurus furcatus* (GCF_023375685.1), *Hemibagrus wyckioides* (GCF_019097595.1), and *Ictalurus punctatus* (GCF_001660625.3).

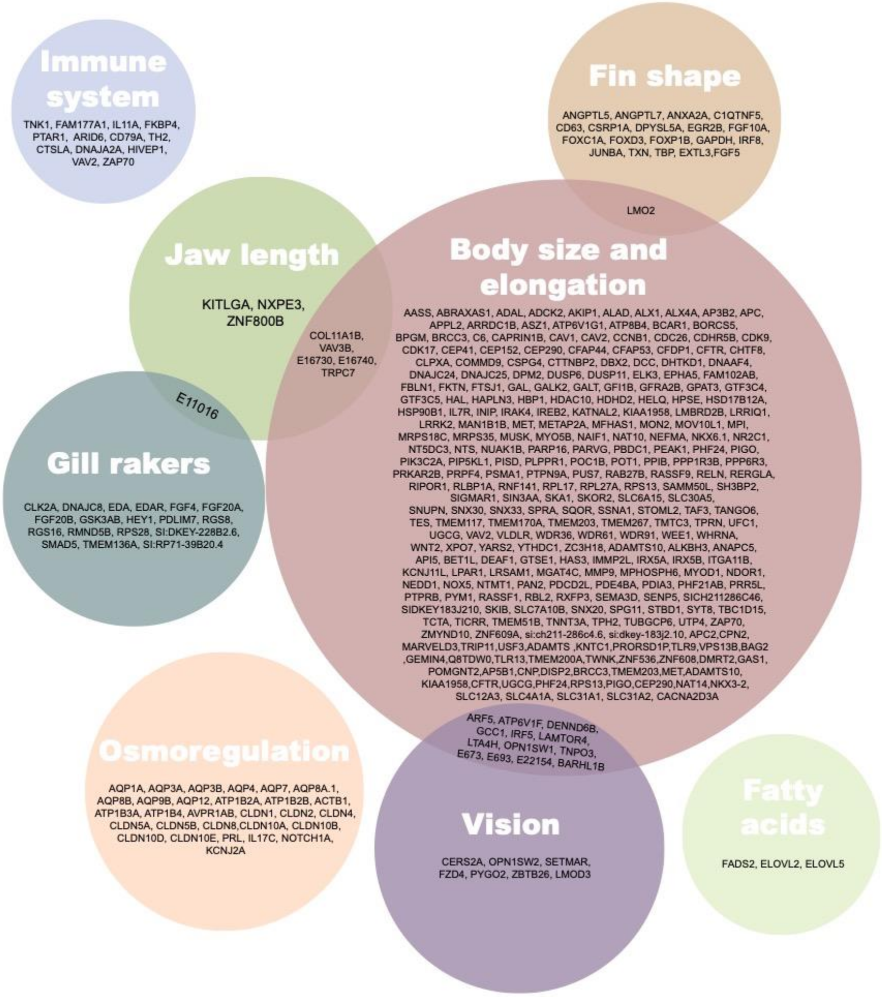

Figure S3. Candidate genes involved in lineage-specific adaptations.

Episodic diversifying selection of candidate and non-candidate genes. To assess the strength of selection at the molecular level using dN/dS and to investigate lineage-specific adaptations in transitions between marine and freshwater habitats, transitions between stenohalinity to euryhalinity, and along the benthic-pelagic axis in the water column, we used various methods from the software package Hypothesis

Testing using Phylogenies (HyPhy v2.5.58[58]). In total, 2,310 genes (249 candidates, 96 BUSCOs, and 1,965 single-copy orthologues) were tested for positive selection using only the master tree due to the computational demands. After applying a BUSTED-E filter to mitigate alignment/sequencing error (see below), we determined the branches of interest for each transition based on the ancestral habitat reconstruction results prior to testing (Figure S4). For marine-to-freshwater transitions, we defined freshwater as foreground branches. For salinity tolerance transitions, we defined euryhaline as foreground branches. For the water-column transitions, we defined pelagic planktivores as foreground branches.

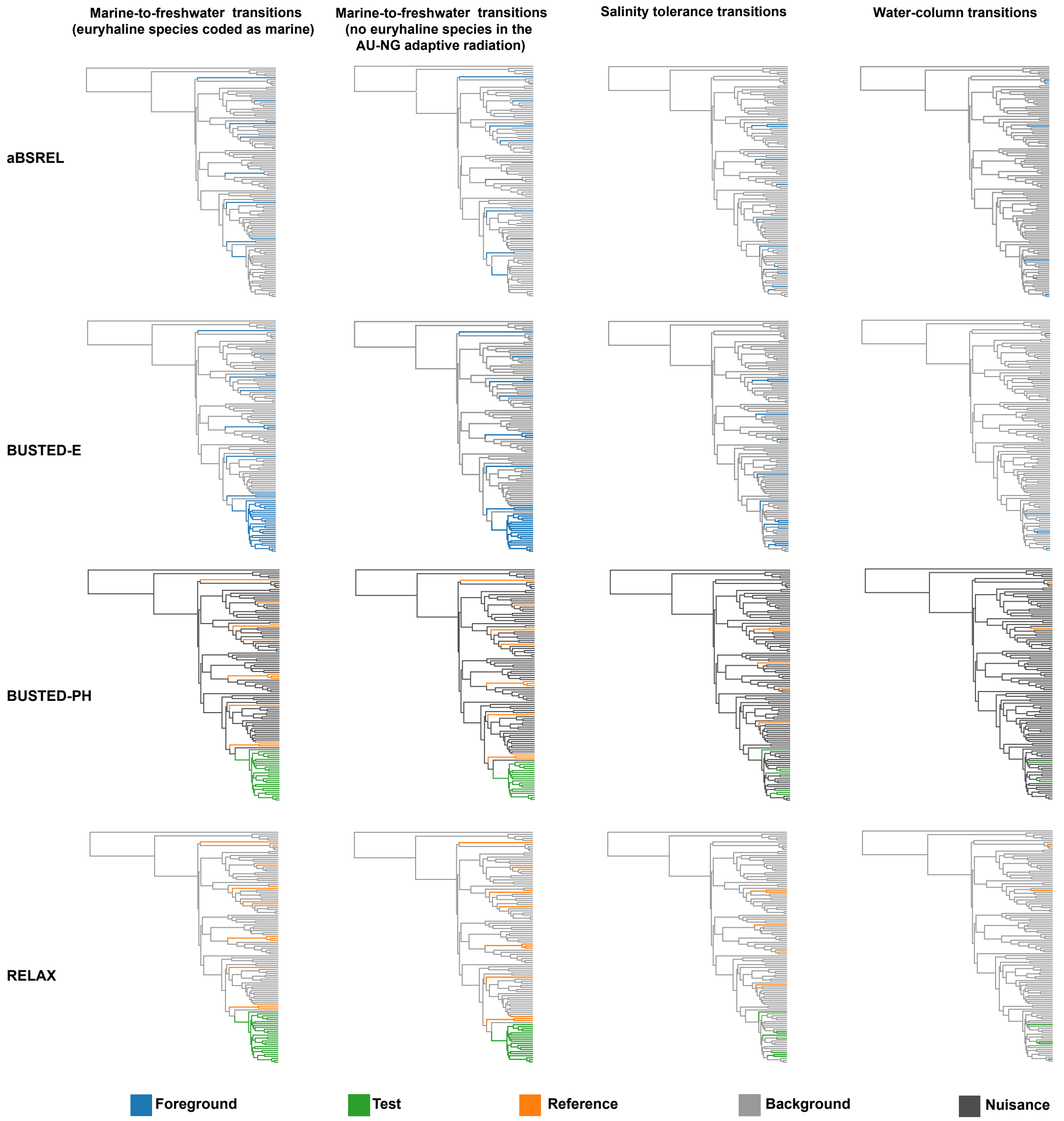

Figure S4. HyPhy schemes used for transitions between marine and freshwater habitats, transitions from stenohalinity to euryhalinity, and along the benthic-pelagic planktivore axis in the water column. In the aBSREL and BUSTED-E methods, foreground branches are depicted in blue. For BUSTED-PH, test branches are depicted in green, while nuisance branches are shown in dark gray. For

RELAX, test branches are displayed in green, reference branches in orange, and background branches in light gray.

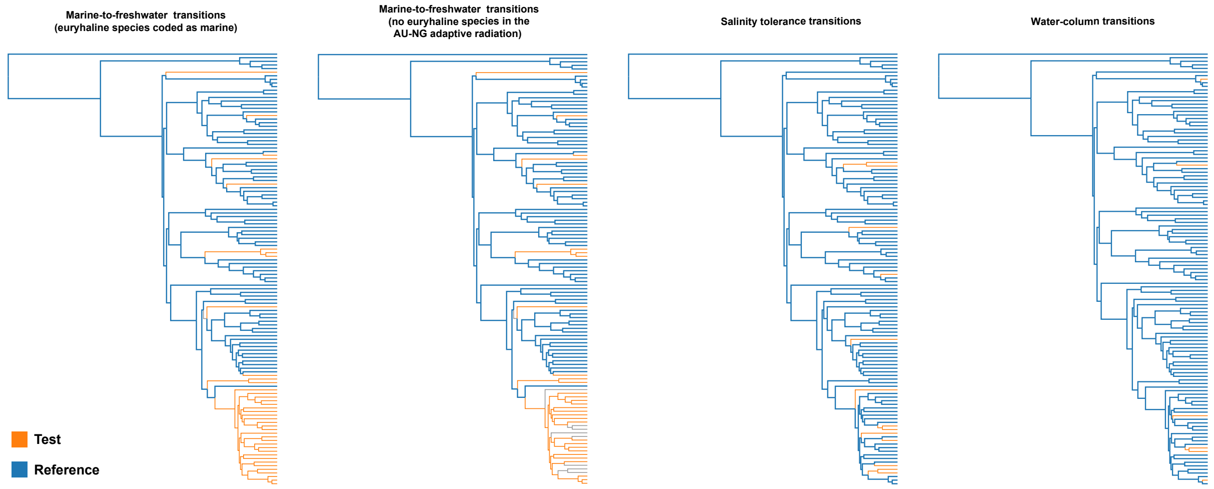

Figure S5. HyPhy schemes used for transitions between marine and freshwater habitats, transitions from stenohalinity to euryhalinity, and transitions along the benthic-pelagic planktivore axis in the water column. Test branches corresponding to species from the derived habitat are depicted in orange, while reference branches corresponding to species from the ancestral habitat are depicted in blue.

The HyPhy analyses for marine-to-freshwater transitions were run in two ways. First, we included all marine-to-freshwater transitions but ignored reversals to marine habitats within AU-NG, a primarily freshwater clade (i.e., we included all species within marine-derived freshwater clades regardless of whether some of them returned to marine habitats). This aligns with the theory on adaptive radiation, which assumes that radiating lineages originate from a common ancestor[59] (the AU-NG radiation is monophyletic), but permits daughter lineages to disperse outside their initial ecological setting (freshwater habitats). In this scheme, euryhaline tips with polymorphic habitat probabilities were coded based on the most likely tip state inferred with SIMMAP. Second, we pruned out euryhaline and marine species within the AU-NG adaptive radiation and repeated the tests.

To determine if a gene experienced positive selection on one or more foreground branches, irrespective of their placement in the tree, we employed the Branch-Site Unrestricted Statistical Test for Episodic Diversification using the new feature –error-sink (BUSTED-E[60,61]). Briefly, this method extends the standard BUSTED-S (with the --error-sink Yes argument) method by adding an “abiological” evolutionary component: branches and sites that evolve with dN/dS ≥ 100, up to 1% total. Local misalignments and mis-annotations which would otherwise trigger positive selection detection are instead captured by this error sink. In this analysis, we designated both stem and crown lineages as the foreground, enabling us to identify selection signatures emerging during or after the inferred habitat transitions. We utilized BUSTED-E to screen the candidate and non-candidate gene sets, comprising a

total of 2,310 genes, to identify regions with potentially abnormal variation patterns indicative of false positive selection signals due to sequencing or alignment errors. Genes tagged as experiencing positive selection with BUSTED-E (119) were retained across various transition schemes for all downstream analyses.

We used the branch-site model with adaptive branch-site random effects likelihood (aBSREL[62]) to identify genes under positive selection during habitat transitions. We focused on the stem lineage of clades featuring the derived habitat state (referred to as foreground clades) to identify genes responsible for immediate effects during the transition. This test interrogates each foreground branch individually, further allowing the detection of signatures of convergence when independent foreground clades exhibit positive selection in the same set of genes.

To investigate whether the evidence of selection was associated with specific phenotypes or traits that might drive adaptation during and after the transitions, we used BUSTED-PHenotype (BUSTED-PH; https://github.com/veg/hyphy-analyses/tree/master/BUSTED-PH). This analysis allowed us to identify genes under positive selection shared between or unique to derived and ancestral habitats, as well as radiating and non-radiating clades. Species inhabiting derived habitats were designated as test branches, while those from ancestral habitats served as reference branches (Figure S5). When comparing radiating and non-radiating derived lineages, we selected four categories of branches for this analysis: test or foreground, representing branches with the phenotype of interest in a focal clade (in this case, AU-NG lineages with the derived habitat state); reference, representing branches with the phenotype of interest in non-focal clades (in this case, remaining worldwide branches with the derived habitat state); background, representing branches without the phenotype of interest (in this case ancestral habitat states); and nuisance, which are not intended for inclusion in the analysis (e.g., reversals to the ancestral habitat state).

We also used RELAX[63] to identify trends and potential shifts (intensification or relaxation) in the stringency of natural selection acting on specific genes. For ancestral versus derived lineage comparisons, we utilized test branches (i.e., lineages inhabiting the derived habitat) and reference branches (i.e., lineages inhabiting the ancestral habitat). To analyze derived lineages, we selected three types of branches: test branches (i.e., AU-NG branches with the target transition), reference branches (i.e., remaining worldwide branches with the target transition), and background branches (i.e., remaining lineages in the tree).

For all HyPhy analyses, we applied a correction to the *p* values by computing a false discovery rate (FDR) using the p.adjust tool and the Benjamini-Hochberg procedure in R. We set an FDR cut-off of 0.05 to determine statistical significance.

Finally, we used the Mixed Effects Model of Evolution (MEME[64]) tool, to analyze the sites under positive selection for a selected number of genes, based on the default significance threshold (*p* value <

0.1). This analysis focused on genes shared among the three transitions that showed the lowest *p* value and were associated with specific functions, including body size and elongation, osmoregulation, fin shape, immune process, erythropoiesis, metabolic process, nervous system, and gill rakers.

Morphological disparity. We conducted a reanalysis of a morphological dataset for ariids, consisting of 28 morphometric (e.g., total length, standard length, eye diameter, interorbital distance) and 2 meristic (i.e., counts of gill rakers and anal fin rays) traits collected from 666 ariid specimens representing 118 species[25]. To evaluate morphological disparity, we employed multivariate trait analysis using the R package dispRity[65]. Our aim was to investigate the impact of habitat on the levels of morphological variation among ariids, focusing on marine-to-freshwater transitions by comparing the levels of disparity between the AU-NG adaptive radiation, freshwater lineages in other regions, and marine lineages. To quantify morphological disparity across habitats, we calculated the sum of variances obtained from the first five principal component scores (~90% of the variance) based on the master tree. Finally, we also examined the potential relationship between morphological disparity and the number of genes under positive selection for body shape and size across the different clades (see below).

Supplementary Notes

Supplementary Note 1

Genome assembly. Our genome assembly for the lesser salmon catfish is 2.34 Gbp, consisting of 372 contigs and 38 scaffolds. The assembly has a %GC content of 42.49 and a N50 scaffold length of 83,992 Mb. The assembly has a total of 28 chromosomes, with 10 scaffolds remaining unassigned. The base calls were of high quality, with an average QV score of 60, indicating a high level of confidence in the sequencing results. The completeness of the Actinopterygii gene set, as assessed by BUSCO, is 97.8%, indicating a high level of completeness. This score is comparable to or higher than the completeness scores typically observed in model organism reference genomes, which are often around or just above 95%[66], even for other catfish genomes (*Ictalurus punctatus*, 96.6%; *I. furcatus*, 95.6%). The reference genome (lesser salmon catfish) contains a substantial number of repetitive sequences, accounting for 72.41% of its composition. Retroelements make up a larger proportion (Class I, 28.35%) compared to DNA transposons (Class II, 24.13%). In comparison, the abundance of retroelements and DNA transposons in other available siluriform genomes is considerably lower[67]. For example, in the genomes of *I. furcatus*, *I. punctatus*, and *Pangasianodon hypophthalmus* retroelements account for 9.12%, 8.24%, and 6.01%, respectively, while transposon characterization contributes 8.45%, 8.95%, and 5.09% to their respective compositions. To enhance the annotation quality being performed by NCBI, we generated

transcriptomic data using PacBio IsoSeq technology. Assembly statistics for the shotgun genome pre-assemblies generated using SPAdes can be found in Supplementary Data S4.

The N50 range for the SPAdes short-read genome pre-assemblies spans from 1.004 Kbp and 3.499 Kbp (Figure S6a). We assessed the quality of the assemblies using the eukaryota dataset in BUSCO v5.4.3[5], resulting in BUSCO-based completeness of less than 20% for all genomes due to the high fragmentation (Figure S7a; Supplementary Data S5).

To address this fragmentation issue, we scaffolded the pre-assemblies using RagTag using the chromosome-level genome of the lesser salmon catfish as the reference. Although the RagTag-based assemblies align with the synteny and number of chromosomes/scaffolds in the reference (not necessarily reflecting the true synteny or number of chromosomes in each species), this scaffolding approach greatly improved the contiguity and accuracy of the genome assemblies, resulting in an overall enhancement in quality. Assembly statistics for the RagTag scaffolding can be found in Supplementary Data S6. The N50 range for RagTag assemblies is between 8.865 Mbp and 67.48 Mbp (Figure S6b). After the scaffolding step, BUSCO-based completeness increased to 78.5-99.6% using the eukaryota dataset (Figure S7b) and 73.2-95% using the actinopterygii dataset (Figure S7c; Supplementary Data S5). The RagTag scaffolding was unsuccessful for *Nemapteryx aff. armiger,* and *Osteogeneiosus militaris* showed a relatively lower N50 value and low BUSCO completeness after RagTag scaffolding. As a result, we relied on the SPAdes pre-assemblies for these particular cases.

Supplementary Note 2

Phylogenomic reconstruction and divergence times. We used the exon capture approach, exon data mined from the shotgun genomes, and legacy markers from previous studies[23–25] to assemble a phylogenomic data matrix consisting of 1,039 exons and 242,949 nucleotide sites for 119 out of approximately 157 species, with 24% data missingness. We conducted phylogenomic analyses using maximum likelihood (ML) and coalescent-based approaches. Inferred trees were resolved with strong support and were largely congruent between subsets and with results from previous studies[23–25,42], providing a robust phylogenomic framework for downstream comparative analyses.

The genus-level taxonomy of ariids adopted in this study follows Marceniuk *et al.*[68], while the subfamily-level taxonomy used follows Acero and Betancur-R.[69] and Betancur-R.[24]. All reconstructions consistently support the division of the family Ariidae into two subfamilies: Ariinae and Galeichthyinae[70]. The subfamily Galeichthyinae, includes four species *Galeichthys ater*, *G. feliceps*, *G. peruvianus*, and *G. trowi*, whereas all remaining species (~97%) belong in the subfamily Ariinae. Within Ariinae, all analyses invariably resolve seven major geographically clustered groups or clades. These include: i) A New World group, a large paraphyletic group that includes several lineages from the genera *Bagre, Notarius,*

Cathorops, Genidens, Sciades, Potamarius, Chinchaysuyoa, and *Ariopsis,* as well as one monotypic genus, *Occidentarius*. ii) An Indo-West Pacific clade comprising species from the genus *Netuma*; iii) A Western Africa clade, which includes species in the genus *Carlarius*. iv) A Madagascar clade, represented by *Betancurichthys madagascariensis.* v) An India and Southeastern Asia group, which includes the genera *Arius, Osteogeneiosus, Jayaramichthys, Cryptarius, Ketengus, Hemipimelodus, Pseudosciades, Kyataphisa, Hemiarius, Cephalocassis*, and *Batrachocephalus*; vi) Another Indo-West Pacific clade containing species from the genus *Plicofollis*; and vii) the Australia and New Guinea adaptive radiation, comprising the genera *Cinetodus, Neoarius, Nemapteryx, Pachyula, Doiichthys, Nedystoma, Paracinetodus, Potamosilurus, Aceroichthys, Pararius, Megalosciades, Pauparius, Cochlefelis, Bleekeriella*, and *Brustiarius*.

We also inferred three additional trees for topology comparison purposes: a concatenation-based ML tree and a species tree using the 432 single copy orthologues from the Actinopterygii OrthoDB, as well as a species tree that reconciles gene duplications using the STAG approach in OrthoFinder based on 3,551 single- and multi-copy BUSCO sequences. The relationships estimated with these approaches were highly consistent with those based on the expanded matrix (Figure S8; Supplementary Data S7).

The inferred dates from subsets with age estimates for MCMCTree analyses are provided in Supplementary Data S8, Figure 1, and Figure S9. Divergence-time estimates are reasonably in good agreement with the age of the ariid stem, as estimated using legacy markers[25]. Crown Ariidae was estimated to be middle Eocene in age (~48.3 Ma, 95% HPD: 44.5-52.3 Ma), whereas the stem age (= crown Anchariidae + Ariidae) was estimated to be around 72 Ma. The subfamily Ariinae emerged in the Oligocene (~31 Ma, 95% HPD: 27-35), and the origin of Old World ariines date to the Miocene (~22 Ma, 95% HPD: 19-26). The Western Africa clade (*Carlarius*) and the Madagascar species (*Betancurichthys*) were estimated to have originated in the Burdigalian of the Early Miocene, around 18 and 20 million years ago, respectively. Similarly, the India-SE Asia clade was estimated to have emerged during the Early Miocene, specifically in the Aquitanian (~20.8 Ma, 95% HPD: 17.8-24.1). Finally, the AU-NG adaptive radiation was estimated to have taken place in the Miocene, approximately ~12.95 Ma (95% HPD: 11-15).

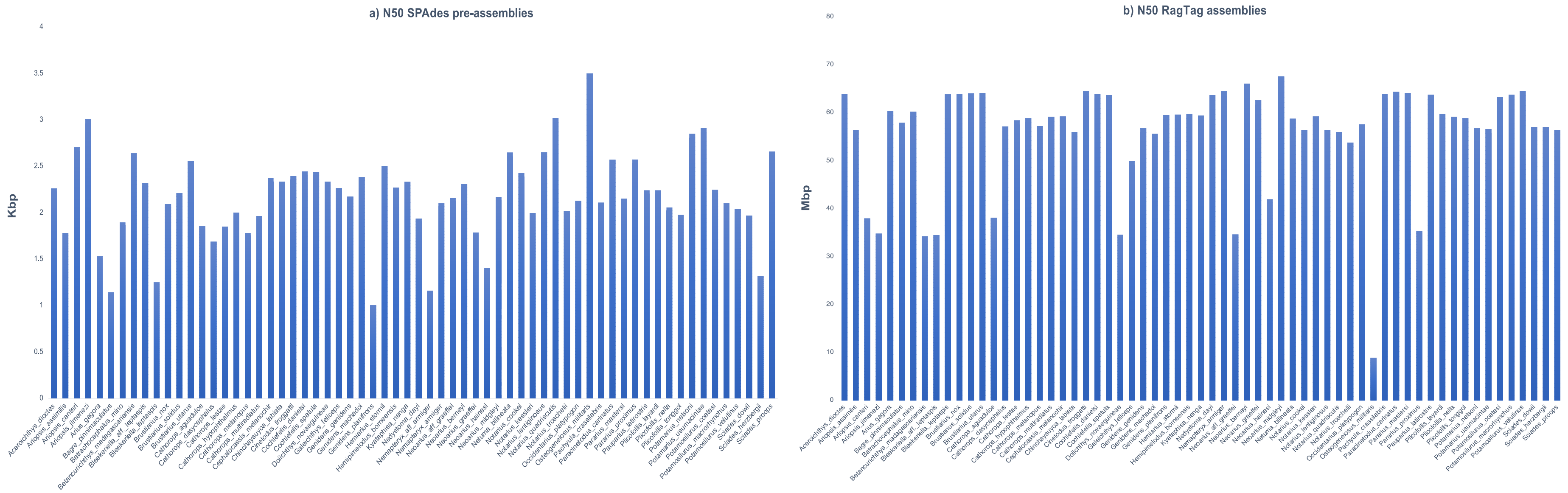

Figure S6. Summary statistics of N50 for the a) SPAdes pre-assemblies and b) RagTag assemblies for the 65 ariid genomes sequenced. N50 values range between 1 Kbp for *Genidens planifrons* and 3.49 Kbp for *Osteogeneiosus militaris* for the SPAdes pre-assemblies, and between 8.86 Mbp for *Osteogeneiosus militaris* and 67.8 Mbp for *Neoarius midgleyi* for the RagTag assemblies. Scaffolding of genomes using a reference genome of the closest related species with short divergence time can generate longer contiguous genomic sequences. Note that plots (a) and (b) have different scales (Kbp and Mbp, respectively).

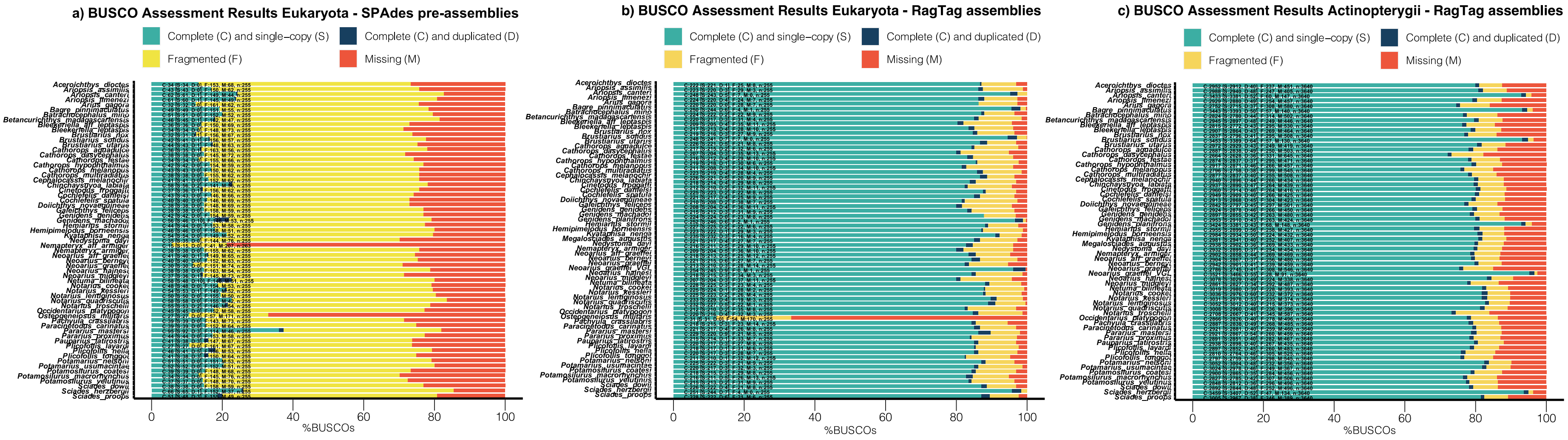

Figure S7. Genome assembly completeness assessed through BUSCO scores. The bar chart illustrates the proportions of complete single copy (S, green), complete duplicated (D, dark blue), fragmented (F, yellow), and missing (M, red) genes. a) The completeness percentages, based on the eukaryote odb10, for the SPAdes pre-assemblies. The SPAdes pre-assemblies exhibit high fragmentation, with completeness ranging from 6% to 37%. b) The completeness percentages are shown for the RagTag assemblies, based on the eukaryote odb10. Out of the 65 genome sequences, 54 had completeness above 80%, while the remaining samples had completeness between 75% and 79%. The specific species falling in this range include *Ariopsis assimilis, Cathorops dasycephalus, Cathorops festae, Cathorops melanopus, Galeichthys feliceps, Nedystoma dayi, Neoarius midgleyi, Plicofollis layardi,* and *Plicofollis tonggol*. Scaffolding failed for *Osteogeneiosus militaris*, for which the completeness was of 12% c) The completeness percentages using the actinopterygii odb10. Among the 65 genome sequences, 26 had completeness between 73% and 79%, 30 had completeness between 80% and 84%, and 6 had completeness between 94% and 95%. The genome of *Nemapteryx aff. armiger* could not be scaffolded with RagTag. The completeness of the reference genome is reported in panels b and c as *Neoarius graeffei* VGL.

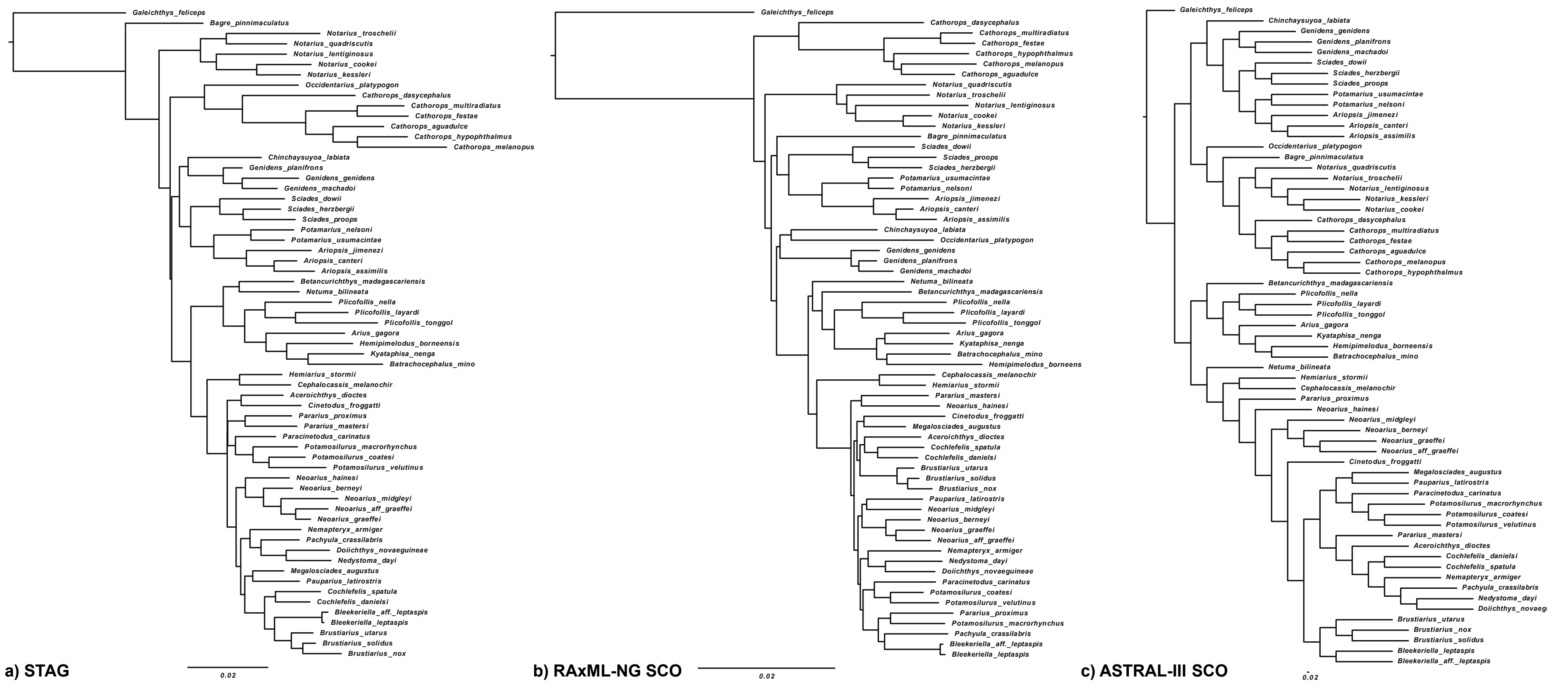

Figure S8. Alternative trees. a) Species tree inferred with the STAG method implemented in OrthoFinder using 3,551 single- and multi-copy BUSCO sequences from the Actinopterygii OrthoDB. b) Phylogenetic tree inferred with RAxML-NG using 432 single copy orthogroups. c) Phylogenetic tree inferred with ASTRAL-III using 432 single copy orthogroups.

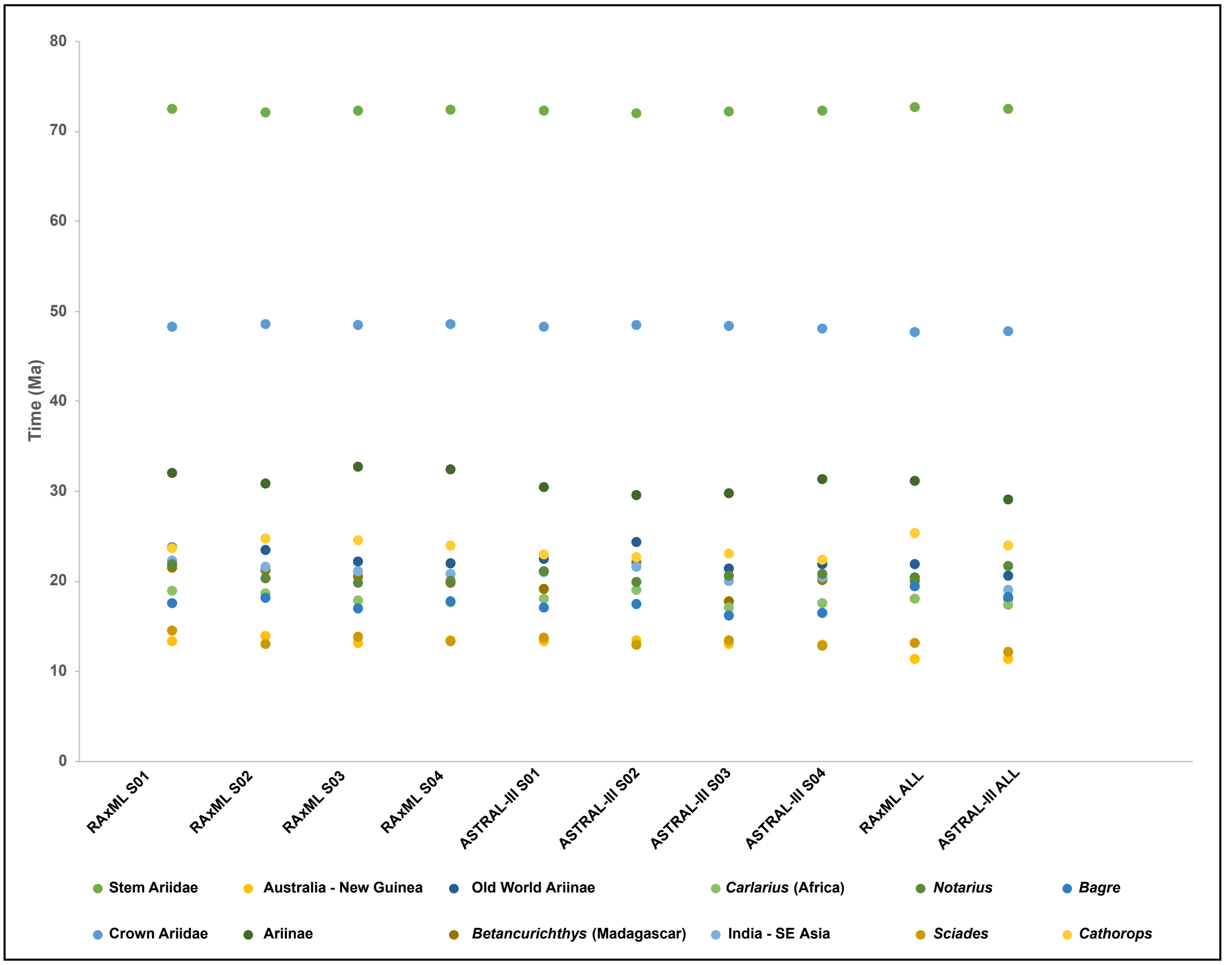

Figure S9. Divergence date uncertainty for major ariid clades based on the 10 trees dated with MCMCTree. 'Master tree': RAxML ALL.

Supplementary Note 3

Habitat transitions. For each dataset, we conducted three independent analyses by assigning species to major habitat categories: i) marine or freshwater; ii) stenohaline or euryhaline; iii) benthic or pelagic planktivore. As noted above, our reconstructions of ancestral habitats account for cases with uncertain habitat occupancy. The ER model was found to have the best fit for transitions between marine and freshwater habitats. For transitions between stenohalinity to euryhalinity, and transitions along the benthic-pelagic axis in the water column, the ARD model had the best fit.

For marine-to-freshwater transitions, our analyses are consistent with previous work[24,25], showing that marine habitats represent the most likely ancestral condition for ariids. We have identified 10-12 transitions to freshwater habitats across various regions worldwide using subset trees, the master tree, and the alternative tree (Figure 1, Figure S10). Furthermore, these trees reveal that 2-3 lineages within the

AU-NG adaptive radiation had reverted to the ancestral marine condition. Additionally, we calculated the average time spent in each state on the complete tree, which revealed that ancestral ariid lineages spent approximately 78% of their time as marine (82 species), while derived ariid lineages spent around 22% of their time as freshwater (37 species) (Supplementary Data S9). For HyPhy trees based on fewer species, for which whole genome data is available and where the sampling is more biased towards freshwater species, ancestral ariid lineages spent approximately 61% of their time as marine, while derived ariid lineages spent approximately 39% of their time as freshwater.

For salinity tolerance transitions, we identified 9-11 shifts towards euryhalinity in diverse biogeographic settings, of which 5-7 occurred within the AU-NG adaptive radiation (Figure 1, Figure S11). Ancestral ariid lineages spent on average, 86% of their time as stenohaline, while derived ariid lineages spent around 14% of their time as euryhaline (Supplementary Data S9). When considering only the genomic data used for the HyPhy analyses, ancestral ariid lineages spent approximately 80% of their time as stenohaline, while derived ariid lineages spent approximately 20% of their time as euryhaline. Regarding water-column transitions, we identified 5-6 transitions to pelagic planktivory (Figure 1, Figure S12). Among these transitions, 3-4 occurred in the AU-NG adaptive radiation, while 2 were observed in in marine species from South and Central America. Ancestral ariid lineages spent on average 95% of their time as benthic, while derived ariid lineages spent on average 5% of their time as pelagic planktivore s (Supplementary Data S9). Focusing solely on the genomic sampling used for the HyPhy analyses, ancestral ariid lineages spent approximately 83% of their time as benthic, whereas derived ariid lineages spent roughly 17% of their time as pelagic planktivores.

Supplementary Note 4

Candidate and non-candidate gene analyses. To investigate transition-specific gene adaptations in freshwater, euryhaline, and pelagic planktivore lineages, we employed the aBSREL, BUSTED-E, BUSTED-PH, and RELAX methods in HyPhy. Refer to the Materials and Methods section above for a description of the various methodologies employed and how they relate to the hypotheses under investigation. Using the master tree, we analyzed a set of 2,310 genes, which comprised 249 candidate genes obtained from the literature, 96 single-copy genes from the Actinopterygii OrthoDB, and 1,965 single-copy orthologs shared among seven Siluriformes species (Figure 2; Supplementary Data S10). We focused on genes that showed a significant p value (FDR corrected p value of <0.05) and were consistently identified across multiple analyses and trees. This approach allowed us to explore positive selection across different analyzes and retain genes that consistently exhibited significance in each habitat transition. We further investigated the overall functional categories of the 2,310 genes using PANTHER.

This analysis revealed significant overrepresentation in twenty-one categories, including cellular process, metabolic process, and biological regulation, among others (Figure S13).

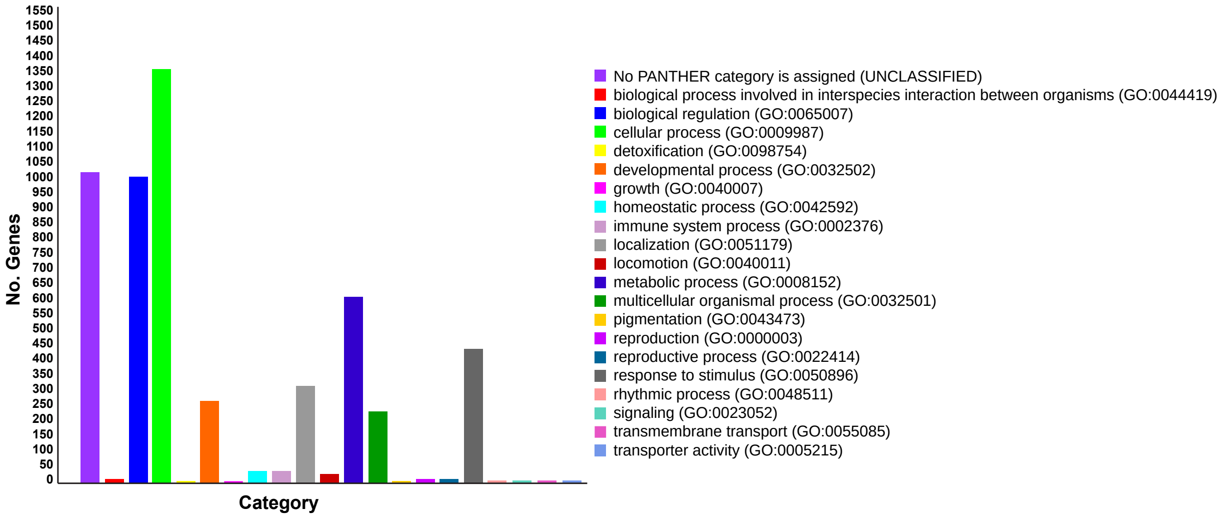

Figure S13. PANTHER bar chart displaying the 21 biological processes associated with the 2,310 candidate, single-copy orthologues, and BUSCO genes.

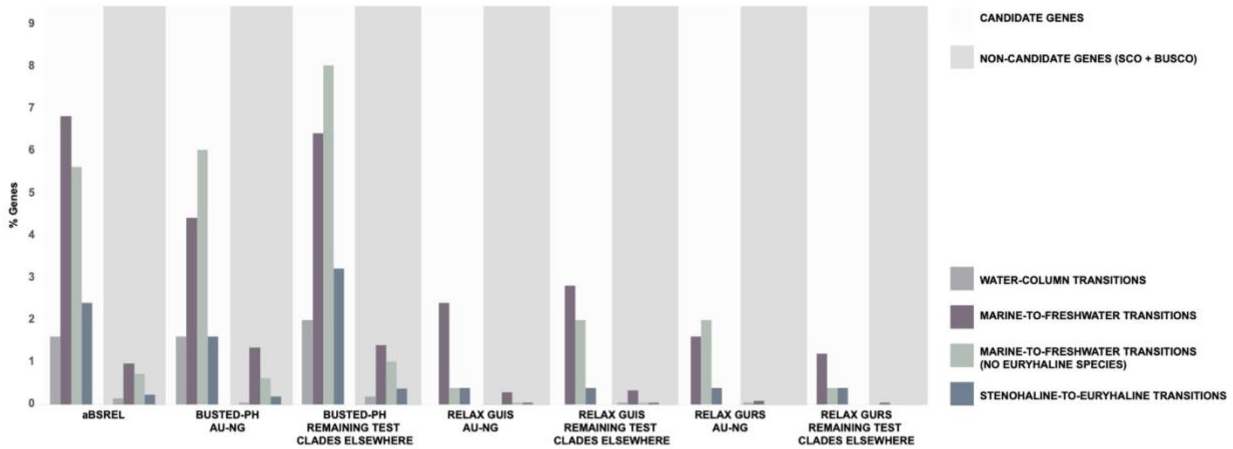

Figure S14. Bar chart displaying the percentage of positively selected genes (PSGs) across different habitat transitions, along with the corresponding datasets. The candidate genes dataset showed a higher percentage of PSGs than the non-candidate genes dataset for all the HyPhy methods tested and the different habitat transitions.

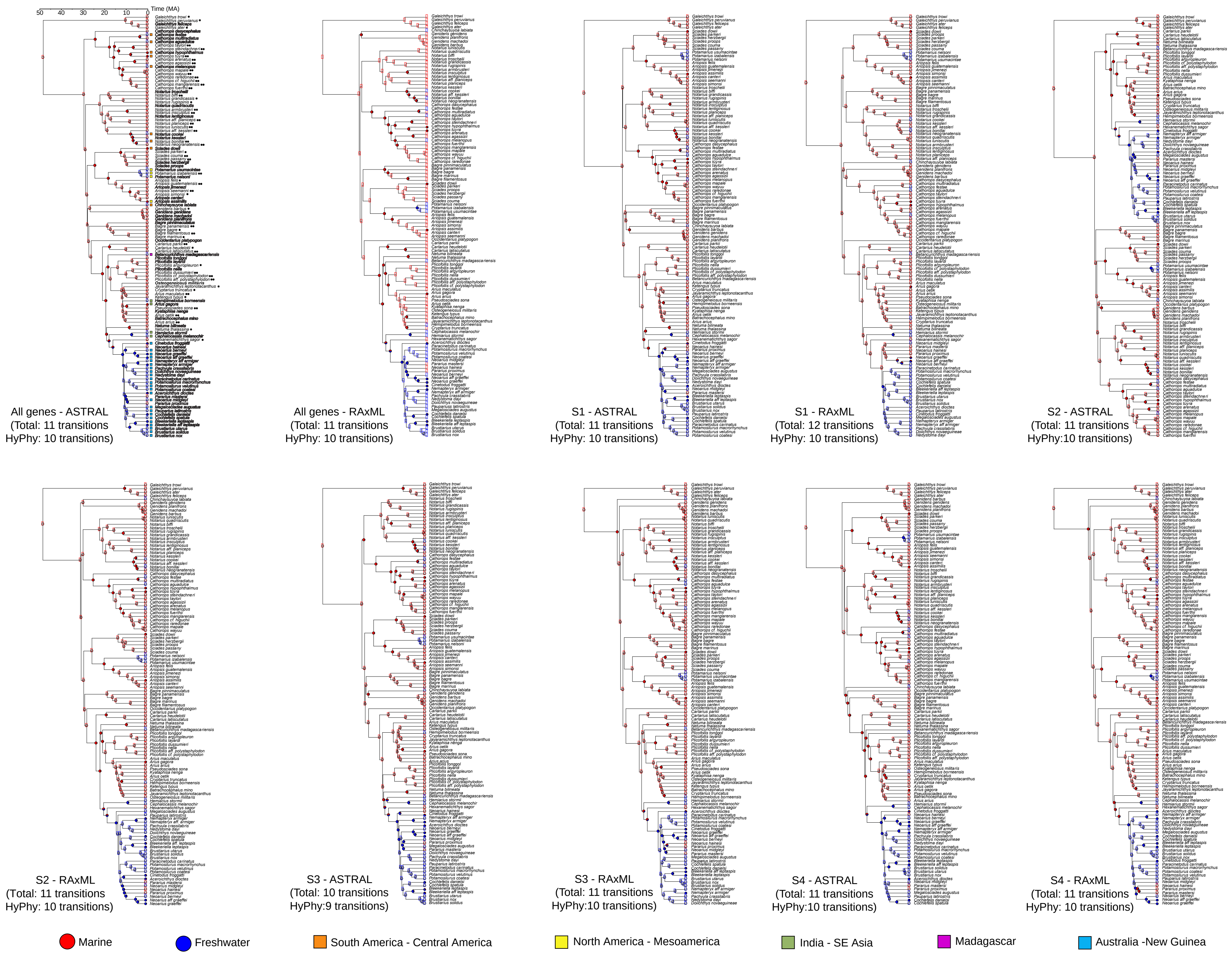
Time (MA)
50 40 30 20 10 0
All genes - ASTRAL
(Total: 11 transitions
HyPhy: 10 transitions)
All genes - RAxML
(Total: 11 transitions
HyPhy: 10 transitions)
S1 - ASTRAL
(Total: 11 transitions
HyPhy: 10 transitions)
S1 - RAxML
(Total: 12 transitions
HyPhy: 10 transitions)
S2 - ASTRAL
(Total: 11 transitions
HyPhy:10 transitions)
S2 - RAxML
(Total: 11 transitions
HyPhy: 10 transitions)
S3 - ASTRAL
(Total: 10 transitions
HyPhy:9 transitions)
S3 - RAxML
(Total: 11 transitions
HyPhy:10 transitions)
S4 - ASTRAL
(Total: 11 transitions
HyPhy:10 transitions)
S4 - RAxML
(Total: 11 transitions
HyPhy: 10 transitions)
Marine
Freshwater
South America - Central America
North America - Mesoamerica
India - SE Asia
Madagascar
Australia -New Guinea

Figure S10. Phylogenomic trees and ancestral habitat reconstruction based on SIMMAP analyses. Trees were estimated using the complete dataset with all 1,039 genes and also four subsets with 265 genes (all subsets overlap in seven legacy markers only: 12S, 16S, ATP6&8, CYTB, MYH6, RAG1, RAG2). Tree inference is based on either concatenation ML analyses (RAxML) or multi-species coalescent analyses (ASTRAL-III). Resulting topologies were time-calibrated in MCMCTree using 14 calibration points. SIMMAP reconstructions (equal rates model) are shown as red (marine) and blue (freshwater) pies for all 10 trees. Marine-to-freshwater transitions are counted when a nodal pie is >50% red and one of its descendant branches is >50% blue. The number of transitions for HyPhy analyses is less than the total number of transitions counted due to fewer species with genomic data available. For the first tree only, tips sequenced based on whole genomes are shown in bold; tips sequenced via exon capture are denoted with one asterisk (*); and tips denoted with two asterisks (**) indicate placement based on the seven legacy markers only (HyPhy analyses used tips in bold only).

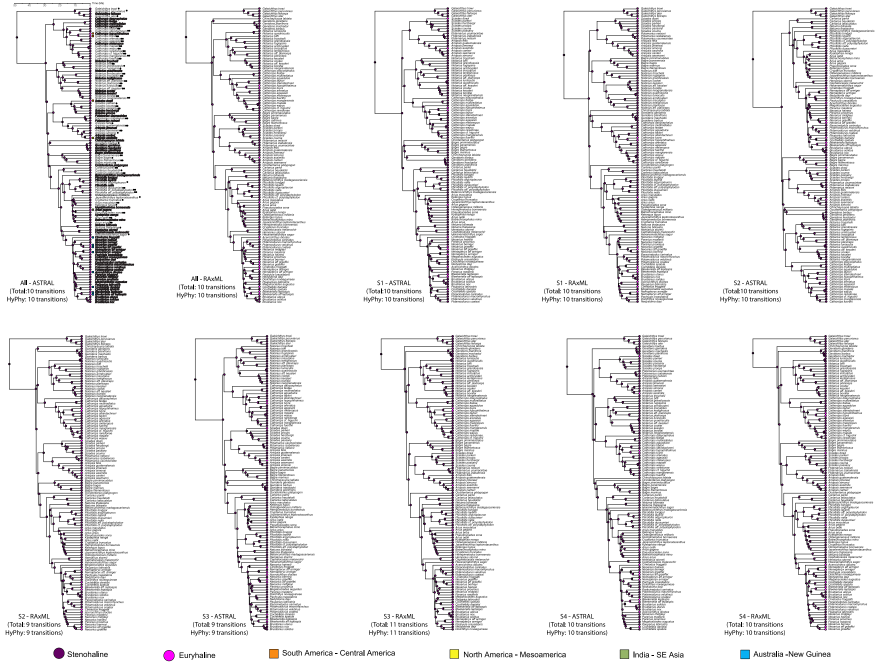

All - ASTRAL
(Total:10 transitions
HyPhy: 10 transitions)
All - RAxML
(Total: 10 transitions
HyPhy: 10 transitions)
S1 - ASTRAL
(Total:10 transitions
HyPhy: 10 transitions)
S1 - RAxML
(Total: 10 transitions
HyPhy: 10 transitions)
S2 - ASTRAL
(Total:10 transitions
HyPhy: 10 transitions)
S2 - RAxML
(Total: 9 transitions
HyPhy: 9 transitions)
S3 - ASTRAL
(Total: 9 transitions
HyPhy: 9 transitions)
S3 - RAxML
(Total: 11 transitions
HyPhy: 11 transitions)
S4 - ASTRAL
(Total: 10 transitions
HyPhy: 10 transitions)
S4 - RAxML
(Total: 10 transitions
HyPhy: 10 transitions)
Stenohaline
Euryhaline
South America - Central America
North America - Mesoamerica
India - SE Asia
Australia -New Guinea

Figure S11. Phylogenomic trees and ancestral habitat reconstruction based on SIMMAP analyses. Trees were estimated using the complete dataset with all 1,039 genes and also four subsets with 265 genes (all subsets overlap in seven legacy markers only: 12S, 16S, ATP6&8, CYTB, MYH6, RAG1, RAG2). Tree inference is based on either concatenation ML analyses (RAxML) or multi-species coalescent analyses (ASTRAL-III). Resulting topologies were time-calibrated in MCMCTree using 14 calibration points. SIMMAP reconstructions (equal rates model) are shown as purple (stenohaline) and magenta (euryhaline) pies for all 10 trees. Euryhaline transitions are counted when a nodal pie is >50% red and one of its descendant branches is >50% blue. For the first tree only, tips sequenced based on whole genomes are shown in bold; tips sequenced via exon capture are denoted with one asterisk (*); and tips denoted with two asterisks (**) indicate placement based on the seven legacy markers only (HyPhy analyses used tips in bold only).

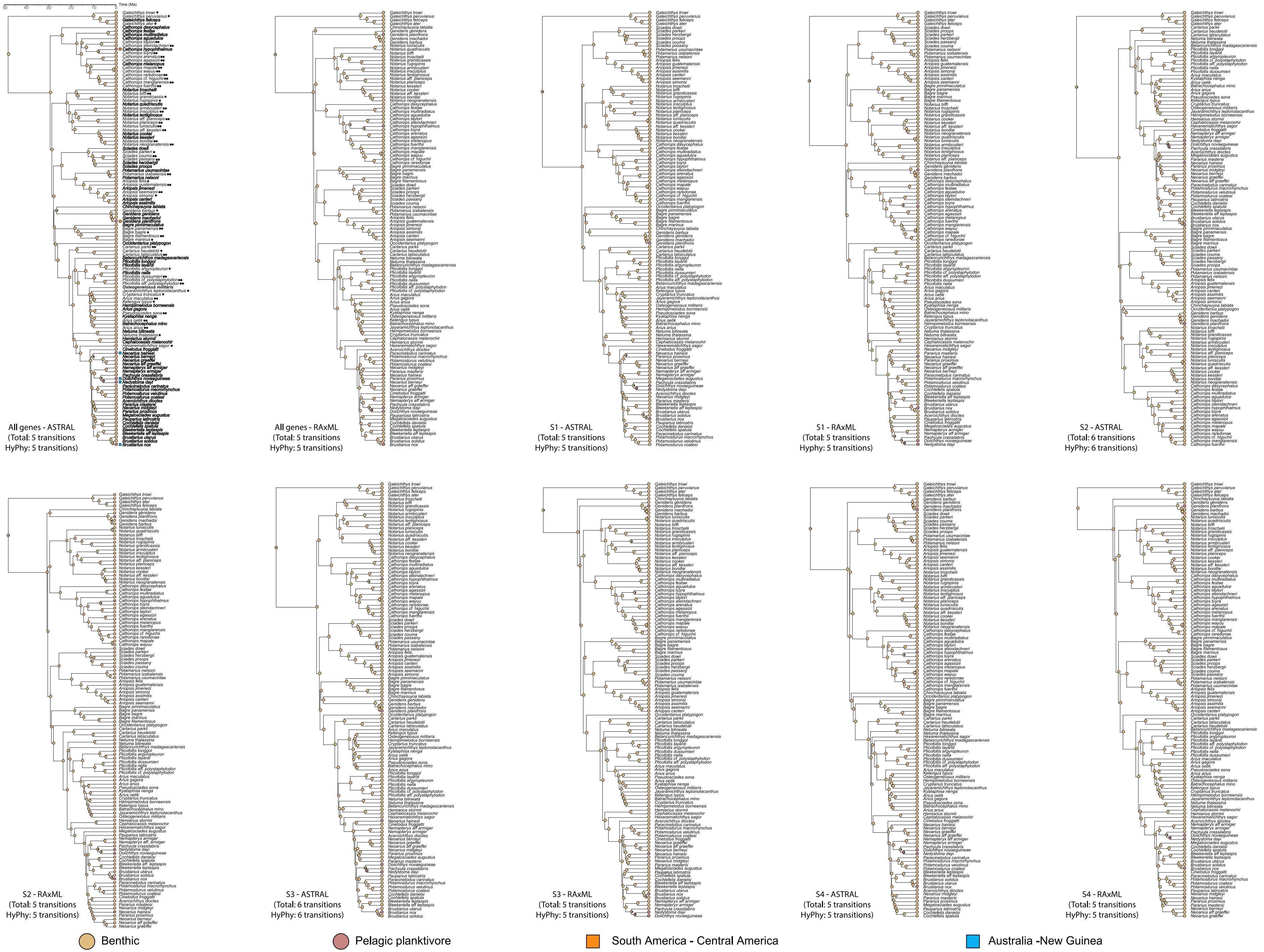
All genes - ASTRAL
(Total: 5 transitions
HyPhy: 5 transitions)
All genes - RAxML
(Total: 5 transitions
HyPhy: 5 transitions)
S1 - ASTRAL
(Total: 5 transitions
HyPhy: 5 transitions)
S1 - RAxML
(Total: 5 transitions
Hyphy: 5 transitions)
S2 - ASTRAL
(Total: 6 transitions
HyPhy: 6 transitions)
S2 - RAxML
(Total: 5 transitions
HyPhy: 5 transitions)
S3 - ASTRAL
(Total: 6 transitions
HyPhy: 6 transitions)
S3 - RAxML
(Total: 5 transitions
HyPhy: 5 transitions)
S4 - ASTRAL
(Total: 5 transitions
HyPhy: 5 transitions)
S4 - RAxML
(Total: 5 transitions
HyPhy: 5 transitions)
Benthic
Pelagic planktivore
South America - Central America
Australia -New Guinea

Figure S12. Phylogenomic trees and ancestral habitat reconstruction based on SIMMAP analyses. Trees were estimated using the complete dataset with all 1,039 genes and also four subsets with 265 genes (all subsets overlap in seven legacy markers only: 12S, 16S, ATP6&8, CYTB, MYH6, RAG1, RAG2). Tree inference is based on either concatenation ML analyses (RAxML) or multi-species coalescent analyses (ASTRAL-III). Resulting topologies were time-calibrated in MCMCTree using 14 calibration points. SIMMAP reconstructions (equal rates model) are shown as amaranth (benthic) and pink (pelagic planktivore) pies for all 10 trees. Benthic-to-pelagic planktivore transitions are counted when a nodal pie is >50% amaranth and one of its descendant branches is >50% pink. For the first tree only, tips sequenced based on whole genomes are shown in bold; tips sequenced via exon capture are denoted with one asterisk (*); and tips denoted with two asterisks (**) indicate placement based on the seven legacy markers only (HyPhy analyses used tips in bold only).

Positive, intensified, and relaxed selection linked to ecological transitions

We compiled two sets of genes: candidate (249 genes), encompassing eight different functional categories in fishes[47,49,51,71–75,57] (see Materials and Methods and Figure S3), and non-candidate (2,061 genes), which included high-confidence single-copy genes from the BUSCO Actinopterygii OrthoDatabase and single-copy orthologues (SCOs) from OrthoFinder[34]. We used these gene sets (total of 2,310 genes) along with a suite of packages (e.g., aBSREL, BUSTED-E, BUSTED-PH, and RELAX) implemented in HyPhy v2.5.58 (see Materials and Methods), to investigate episodic diversifying selection associated with transition-specific signatures in freshwater, euryhaline, and pelagic planktivore lineages across Ariidae. See Figure 2 and Figures. S15-S18.

We first used BUSTED-E to filter the gene sets by identifying regions that may display unusual variation patterns (e.g., w > 100 and weight < 1%), which could indicate potential false positive signals of selection stemming from sequencing or alignment errors. An initial comparison between BUSTED-PH/aBSREL vs. BUSTED-E using the treatments explaining above, shows a large difference in the number of genes under positive selection identified by these approaches (84, 189, 26 respectively), suggesting that failure to correct for alignment/sequencing errors could significantly inflate the number of genes detected to be under positive selection (Figure S14). We thus retained the 119 BUSTED-E genes across various transition schemes for all downstream analyses.

We then assessed whether the candidate genes selected *a priori*, which were identified by previous studies[47,49,51,71–75,57] to have an association with traits involved in fish adaptations, had a higher incidence of positive selection relative to the non-candidate BUSCOs and SCOs gene sets. Across all transitions examined, candidate genes exhibited a higher proportion of PSGs compared to non-candidate genes (0.4-8% vs 0.04-1.4%, respectively; Figure S14).

Overall, marine-to-freshwater transitions showed the highest number of genes under positive selection, followed by stenohaline-to-euryhaline and water-column transitions. However, this trend is influenced in part by the number of branches tested within each habitat transition (Figure 2; Figure S4), and thus are not necessarily comparable. Across these transitions, genes associated with ecological specialization, including those involved in developmental, metabolic, immune, reproductive, and transport processes, consistently experienced positive diversifying selection (see Figure 2). Additionally, we found 21 significantly overrepresented biological process Gene Ontology (GO) terms across all transitions (Figure S13; Supplementary Data S10).

The number of PSGs is influenced by the duration that lineages spend in different habitats, which can be misleading when comparing habitats or clades. Ariids have predominantly occupied ancestral habitats (marine, stenohaline, benthic) for 78-94% of their evolutionary history when considering complete taxonomic sampling, and 61-84% when considering trees with whole-genome species only that

were used for positive selection analyses (Supplementary Data S9). This contrasts with the limited time spent in derived habitats (freshwater, euryhaline, pelagic-planktivore), ranging from 5-22% (complete taxonomic sampling) to 16-39% (whole-genome sequences) (Supplementary Data S9). Our results reported below take these factors into account. Additionally, based on the two alternative coding schemes implemented for marine-to-freshwater transitions (see below and Supplementary Materials and Methods), our analyses reveal that HyPhy can be sensitive to tree choice. Initial analyses based on the master tree only show significant differences in PSGs for the marine-to-freshwater transition, depending on how species are coded (e.g., 37, 39, 45 with euryhaline species; 14, 28, 41 without euryhaline species for aBSREL, BUSTED-PH AU-NG, and BUSTED-PH remaining test clades; see Supplementary Data S15a). However, when analyses are performed using the 10 trees and only PSGs identified in at least nine trees are selected, the differences become more subtle (e.g., 40, 64, 59 with euryhaline species; 39, 45, 51 without euryhaline species for aBSREL, BUSTED-PH AU-NG, and BUSTED-PH remaining test clades; see Supplementary Data S15b). Thus, all results reported below are based on a 9+ trees cutoff.

Supplementary Note 5

Marine-to-freshwater transitions. For this analysis, we used two coding strategies. First, we coded euryhaline species based on the most probable tip state (either freshwater or marine) based on SIMMAP, which accounted for tip-state ambiguity. Following theory of adaptive radiation, we coded all tips within the AU-NG radiation with the ancestral habitat state for the clade, which assumes that radiating lineages originate from a common ancestor[59] (see Supplementary Materials and Methods). The second approach excluded euryhaline species, as well as tips that displayed reversals to the ancestral marine condition within the AU-NG clade (see Supplementary Materials and Methods). The way euryhaline species are coded for marine-to-freshwater transitions has a substantial impact on the results (excluding euryhaline species results in much fewer PSGs than including them; see Supplementary Data S15). Thus, we focus this section on the set of 66 shared PSGs across coding strategies.

Genes associated with body size and elongation exhibited the highest number of PSGs in freshwater lineages. Body shape divergence in fish is one of the most common patterns of morphological variation associated with adaptation to a new environment and habitat specialization. The genetic regulation of body size and shape is a complex trait influenced by multiple genes (highly polygenic) and environmental factors[51,76]. When comparing marine and freshwater lineages, 26 out of 61 PSGs were associated with body size and elongation in freshwater lineages and 22 out of 43 in marine lineages. Although the proportion of PSGs associated with these traits is higher in marine lineages, the total number of relevant PSGs is greater in freshwater lineages. This observation seems to align with the higher phenotypic disparity observed in freshwater lineages (Figures 3, 4; Figure S15-S16). It also underscores a

strong genotype-phenotype connection[77,78]. Similar patterns have been reported in other marine-derived freshwater clades such as Belonidae and Tetraodontidae, where body size disparity is higher in freshwater habitats[79]. PSGs in ariids associated with this category include genes from the Insulin-like growth factor and transforming growth factor-beta gene families, which have also been found in studies with cichlid fishes[51,80]. Other notable PSGs include genes related to development and growth[49,81], such as *CFAP53,* which plays an important role in the very early development of the zebrafish embryo[82], *HBP1,* which regulates the timing of cortical neurogenesis by elongating the cell cycle and is essential for normal cortical development[83], and *LRRK2,* which is involved in development and regeneration[84] (Figures 2, 4).

The category with the second-highest number of PSGs was associated with immune processes, showing a higher number of immune-related PSGs in marine (6) compared to freshwater (4) lineages. Freshwater species exhibit varying immune responses to salinity changes; when marine species shift to freshwater, they encounter unfamiliar pathogens and microbial communities, necessitating immune response genes that regulate immune cell function, pathogen recognition, and cytokine production[85,86]. Notable PSGs in this category include genes such as *TNK1*, involved in innate immunity[87], *IL-11*, associated with immune cell activation[88], *SGPL1*, linked to immune processes[89], *TH2*, essential for maintaining immune homeostasis and combating parasitic infections[85], and *DNAJA2*, involved in developmental stages and stress responses[90].

Additional PSGs associated with this transition in other functional categories include genes related to the osmoregulatory system, such as aquaporin 7 (*AQP7*) a water channel protein linked to cell volume regulation and sensing[73], and prolactin (*PRL*), known for its role in regulating chloride cells with a gradual action[91]. Vision-related genes, including *PYGO2*, involved in the development and function of the visual system in fishes[92], appeared to be under positive selection among all freshwater species. Additionally, variations in fin-shape genes can be attributed to changes in the regulation of fin ray growth, which involve processes such as cellular proliferation, differentiation, and survival[80]. PSGs related to fin shape include the fibroblast growth factor family[93] and exostosin-like glycosyltransferase 3 (*EXTL3*), known to affect cartilage development and pectoral fin formation[94,95].

We aimed to assess the impact of positive selection during and after the transition to freshwater habitats. We observed a smaller number of PSGs directly involved in the transition (37) compared to those identified during and after the transition (66). During habitat transitions, organisms initially rely on pre-existing genetic variation to cope with the new environment, requiring fewer new genetic adaptations[96,97]. As they acclimate and establish themselves, increased positive selection on a broader range of genes occurs, reflecting a dynamic interplay between genetic adaptation and environmental change.

These PSGs were associated with various biological functions, including biological regulation, cellular processes, developmental processes, homeostatic processes, immune system responses, multicellular organismal processes, cellular localization, response to stimuli, and transmembrane transport. We found some PSGs closely linked to traits such as body size and elongation (e.g., *ATP6V1G1, CDK17, CFAP53*), and immune function (e.g., *HIVEP*). Additionally, we identified PSGs acting during the transition related to other traits such as fin morphology (e.g., *EXTL3, FOXC1A, FGF4*), erythropoiesis (*ADNP2*), and osmoregulation (*PRL*), (Figure 2, Figure S17). Further details on the osmoregulatory gene are provided below.

We also conducted a site-specific positive selection test to determine the contribution of PSGs to convergent and parallel amino acid substitutions. Using the mixed effects model of evolution MEME, we analyzed 66 PSGs and identified 663 PSSs associated with transitions from marine to freshwater environments, indicating significant evolutionary pressure on these genes during adaptation (Supplementary Data S16). The PSSs were primarily linked to genes involved in cellular processes (30), followed by those related to biological regulation (22), developmental processes (10), response to stimulus (9), localization (4), and immune processes (3). Looking at parallel (identical substitutions) vs. convergent (different substitutions) changes in PSSs, our results did not reveal ecological PSS convergence or PSS parallelism involving the same mutation at the same amino acid position across all freshwater clades (Figure S18; Supplementary Data S16). However, 396 out of 663 PSSs were shared between at least two freshwater clades, and in the 15 notable PSSs (in 9 PSGs) depicted in Figure S18, 6 display convergence, whereas 12 show parallel changes. These results suggest a balance between common genetic changes and diverse evolutionary paths among different lineages during freshwater adaptation[98,99].

Salinity tolerance transitions. In the transition from stenohalinity (narrow salinity ranges) to euryhalinity (tolerance of a wide range of salinities), our BUSTED-PH analyses revealed a higher number of genes under positive selection for stenohaline lineages (67) compared to euryhaline lineages (33). This pattern was anticipated, given that ariids have spent 80–85% of their time in the stenohaline state and 15–20% in the euryhaline state (Figure 3; Figure S15).

Six genes unique to euryhaline lineages were identified when comparing PSGs between stenohaline and euryhaline lineages, each potentially playing a critical role in adapting to changing environmental conditions. Among them, Kininogen-1 (*KNG1*) is known for its role in regulating blood pressure and inflammation, suggesting it may play a crucial role in environmental adaptation[100]. Ceruloplasmin (*CP*), involved in the immune response and possessing antimicrobial properties, acts as an antioxidant in plasma through its ferroxidase activity, contributing to iron homeostasis[101]. Purine nucleoside phosphorylase (*PNP*) is linked to both immunodeficiency and autoimmunity[102]. GINS

complex subunit 3 (*GINS3*), as part of the GINS heterotetrameric complex, is crucial for DNA replication initiation and replisome progression[103], maintaining genomic integrity during cellular division. SYNDECAN-3's (*SDC3*) involvement in inflammation and angiogenesis suggests a potential role in immune response and tissue remodeling, beneficial in adapting to the changing conditions of euryhaline habitats[104]. Lastly, translocase of inner mitochondrial membrane 29 (*TIMM29*) is involved in protein insertion into the mitochondrial inner membrane, essential for maintain efficient mitochondrial function in fluctuating energy demands of euryhaline habitats. These results suggest that the genes experiencing positive selection may play essential roles in these adaptations, protecting ariids from environmental stressors. These findings are intriguing, as transitions to euryhalinity involve changes in oxygen uptake, metabolism, hydromineral balance, and the immune system, particularly lymphocyte activities and circulating cytokine levels[85].

Only two osmoregulatory genes, *AQP7* and *PRL*, were identified as being under positive selection during salinity tolerance transitions. In contrast, other osmoregulatory-related genes, including claudins, solute carrier proteins, ATPase Na+/K+ transporting, and arginine vasopressin receptors, did not exhibit signs of positive selection. These findings suggest a potential preadaptation, where euryhaline ariids may already possess genetic variations in osmoregulatory genes that facilitate adaptation to a wide range of salinities. Consequently, there may be less selective pressure acting on these genes compared to other functional categories[105].

We then examined the impact of positive selection during and after the transition to euryhaline habitats. We found that a smaller number of PSGs were directly involved in the transition (28) compared to those identified during and after the transition (72). Subsequently, we analyzed these 72 genes using the MEME method to identify amino acid sites under positive selection, revealing 472 PSSs. The PSSs were primarily associated with genes involved in cellular process (31), biological regulation (22), metabolic process (13), developmental process (9), immune process (6), and transmembrane transport (2). Among these 472 PSSs, 58 were shared between at least two euryhaline clades. In Figure S18, we highlight four notable PSSs (in four PSGs), with only one displaying convergence (position 20 of the *SOX21B* gene), while four exhibit parallel changes. This pattern is commonly observed, as close relatives are more likely to share the same ancestral state before independent substitutions occur[106] (e.g., Figure S18, position 99 of the *ADARB1B* gene).

Water column transitions. In our analysis of transitions within the water column, we found 58 PSGs associated with benthic lineages, which have predominantly inhabited this habitat, accounting for 83–95% of their evolutionary history. In contrast, pelagic planktivore species, which have spent only 5–17% of their evolutionary time in this habitat, displayed 13 PSGs (Figure 3).

Among the PSGs unique to pelagic planktivore lineages compared to benthic lineages, three genes stand out. The zinc transporter 10 (*SLC39A10*), crucial for fetal definitive hematopoiesis by maintaining zinc homeostasis, plays a vital role in hematopoiesis and early development[107]. The DNA primase small subunit (*PRIM1*), responsible for synthesizing short RNA primers necessary for DNA replication, is essential for cell growth, development, and repair, ensuring accurate duplication of genetic material[108]. Additionally, *PNP*, associated with immune processes[102], was identified among these unique PSGs.

In examining amino acid substitutions, we analyzed 75 PSGs and identified 353 PSSs associated with water-column transitions. These PSSs were predominantly linked to genes involved in cellular processes (35), biological regulation (26), metabolic processes (14), response to stimuli (12), developmental processes (10), and transmembrane transport (2). Among these PSSs, 14 were shared between at least two pelagic planktivore clades. In Figure S18, we highlight five notable PSSs (in four PSGs), with two displaying convergence (position 8 of the *FKBP4* gene and position 95 of the *ATP6V1G1* gene), while only one exhibiting two parallel changes (positions 108 and 121 of the *LRSAM* gene).

Supplementary Note 6

Adaptive radiation in Australia & New Guinea vs. other clades in derived habitats elsewhere. When analyzing positive selection results across the three transitions and comparing the AU-NG adaptive radiation with the remaining test clades, we observed a higher incidence of PSGs in non-radiating clades compared to the AU-NG adaptive radiation across all transitions (Supplementary Data S14-S15). This difference may be attributed to the presence of more lineages outside the adaptive radiation and the longer time these species have spent in the non-radiating habitat compared to their radiating counterparts (Figure 1). Functional categories that exhibited the highest count of PSGs in both radiating and non-radiating clades included cellular processes, biological regulation, metabolic, developmental, response to stimulus, immune, and transmembrane transport (Figures S15- S16).

During transitions involving the water column and salinity tolerance, genes associated with body size and elongation comprised the largest proportion of PSGs in the AU-NG adaptive radiation (50% and 44% respectively). However, in marine-to-freshwater transitions, the percentage of PSGs linked to body size and elongation was higher in the remaining freshwater test clades compared to the AU-NG clade (27% in AU-NG vs. 43% in the remaining test clades). This disparity suggests differing evolutionary pressures or adaptive strategies between these groups during habitat transitions. Genes related to immune processes were the next most abundant category in the AU-NG clade across all three habitat transitions (10 % for water-column transitions, 7% for the marine-to-freshwater transitions, and 5% for the salinity-tolerance

transitions). This suggests potential immune system adaptations to combat pathogens and environmental challenges encountered during habitat colonization[109].

When investigating PSGs in stem lineages associated with habitat shifts using aBSREL, we found that the non-radiating freshwater clades exhibited a higher number of PSGs (32) compared to the AU-NG adaptive radiation (1). In contrast, in salinity-tolerance and water-column transitions, the AU-NG adaptive radiation showed a higher number of PSGs (21, 11, respectively) than the remaining test clades (12, 7). Analyses of positive selection during and after the transition using BUSTED-PH, the remaining test clades consistently showed a higher number of PSGs for the marine-to-freshwater, stenohaline-to-euryhaline and water-column transitions (49, 69, 73) compared to the AU-NG adaptive radiation (41, 18, 10), indicating a consistent trend of higher PSG counts in the remaining test clades across multiple habitat transitions compared to the AU-NG adaptive radiation.

We found that during transitions involving changes in salinity, two osmoregulatory genes, *AQP7* and *PRL*, appeared to have played pivotal roles in adaptation. Interestingly, in the salinity-tolerance transition, these genes underwent positive selection in euryhaline species outside the adaptive radiation, whereas in the marine-to-freshwater transition, they were positively selected within the adaptive radiation. *PRL*, emerged as a significant PSG in the aBSREL results, indicating its involvement in immediate effects during the transition, a hypothesis further supported by MEME analyses (see Figure 4 and Figure S18). Among the 12 PSSs unique to the AU-NG clade, out of the total 663 PSSs associated with marine-to-freshwater transitions, three alone were found on the osmoregulatory *PRL* gene (positions 121, 162-163). Position 121 showed convergence between all AU-NG radiation species and a Neotropical freshwater species (*Chinchaysuyoa labiata*), while positions 162-163 were unique to the AU-NG clade. These results suggest that an osmoregulatory adaptation in prolactin in the ancestor of the AU-NG radiation may have facilitated its successful colonization of freshwater habitats in Australia and New Guinea. Additionally, one PSS was found on two different genes associated with body size and elongation (*DMRT2* and *RERGLA*), displaying parallelism at positions 323 and 336, respectively, among all AU-NG radiation species. These findings underscore the potential significance of these genomic factors in aiding the colonization and diversification of sea catfishes in Australo-Papuan rivers, possibly facilitated by specific pre-adaptations, particularly evident in the PRL gene, with three PSSs identified within the AU-NG clade.

Our study sheds additional light on the complexity of the potential adaptation process from marine to freshwater environments. The involvement of *PRL*, instead of the more commonly implicated aquaporins, claudins, and vasopressin genes found in other fish species[110–112], suggests that multiple pathways may exist for fish to adapt to freshwater habitats from ancestral marine environments[99,113]. This also challenges the deterministic view proposed by Ishikawa *et al.*'s fatty acid metabolic study[75], as we did not find multiple copies of *FADS2*, and the sole copy we identified was not under positive selection

despite the recognized importance of fatty acid metabolism in overcoming nutritional constraints associated with freshwater adaptation in ray-finned fishes. These findings suggest a more nuanced and multifaceted mechanism underlying the adaptation to freshwater environments.

As previously noted, transitions along the benthic-to-pelagic axis are significant drivers of divergence in fish radiations, often exerting selective pressure on genes related to body depth[51] (i.e., *ATP6V1G1, LRSAM1, SIGMAR1*), fin shape[114] (*EXTL3*), and gill raker development[47,115,116] (*FGF4*), potentially enhancing locomotion and prey capture efficiency. This pattern is evident in various fish radiations, including cichlids, sticklebacks, and whitefishes[46–51]. Despite comprising more pelagic planktivore lineages, the AU-NG adaptive radiation exhibited fewer PSGs (10) for benthic-to-pelagic transitions compared to other pelagic clades elsewhere (73). Our positive selection analyses focused on pelagic planktivore species, specifically highlight one gene associated with gill raker formation[47,49] in the AU-NG adaptive radiation, the Fibroblast Growth Factor family 4 (*FGF4*). Additionally, two other genes, a voltage-dependent calcium channel (*CACNA2D3A*) and the CDC Like Kinase 2 (*CLK2A*), showed signs of positive selection in the remaining pelagic planktivore species. The finding in the *FGF4* gene related to gill rakers was supported by MEME analyses, revealing a single change at position 218 present in *Brustiarius nox*, the ariid species with the highest raker counts (58). This suggests a potential association between genetic variation in *FGF4* and morphological adaptations in gill raker development.

Supplementary Note 7

Strength of selection. Our analyses revealed a prevailing trend of intensified selection in derived (test) habitats or states across the three distinct transitions, relative to their ancestral (reference) counterparts. This suggests that evolutionary pressures associated with these transitions favor the acquisition of novel genetic adaptations.

Freshwater lineages transitioning from marine environments displayed intensified selection on three potentially adaptive genes: odorant receptor 131-2 (*OR131-2*), a kazal-type serine peptidase inhibitor domain 3 (*KAZALD1*), and heme transporter *FLVCR2*. These genes may enhance sensory perception, defense against novel pathogens, and iron metabolism in iron-depleted freshwater habitats[117–119]. In contrast, *DMRT2,* involved in somite development and sex determination[120], exhibited relaxed selection, suggesting a potentially reduced or modified ancestral function in freshwater. Interestingly, genes like *ATP6V1G1* and *TMEM170A*, crucial for pH and calcium homeostasis[91], showed relaxed selection within the AU-NG adaptive radiation but intensification in the non-radiating lineages elsewhere, potentially due to varying environmental factors across different freshwater systems.

Euryhaline lineages across both radiating and non-radiating clades showed intensified selection for four genes compared to stenohaline lineages: *PRIM1* and *PNP*, essential for DNA replication and

nucleotide metabolism[102,108], and *LRRK2* and *NR2C1*, involved in development and regeneration[84]. This highlights adaptations for coping with the challenges of fluctuating salinity environments. The heightened selection on essential cellular processes like DNA replication and nucleotide metabolism, underscores their importance in osmotic stress adaptation, while positive selection on development-related genes may reflect changes in body size and elongation in euryhaline species. Interestingly, sigma non-opioid intracellular receptor 1 (*SIGMAR1*), a multifunctional gene[121], showed relaxed selection in euryhaline lineages. This suggests the presence of potential pre-existing mechanisms for osmotic stress management.

Water column transitions revealed intensified selection in pelagic planktivores compared to benthic species for two genes: *PNP* and *LRSAM1*. This suggests adaptations in key systems like development, immunity, and potentially nervous system function during the shift to a pelagic habitat[102,107]. Interestingly, *PNP* exhibited contrasting patterns of selection between the AU-NG adaptive radiation (GUIS) and the remaining pelagic planktivore test clades (GURS), suggesting distinct evolutionary forces shaping this gene. Conversely, *SLC39A10* showed the opposite pattern, relaxation of selection for the AU-NG adaptive radiation and intensification of selection for the remaining pelagic planktivore test clades. These differing patterns suggest that distinct evolutionary forces shaped these genes under varying circumstances within the pelagic environment.

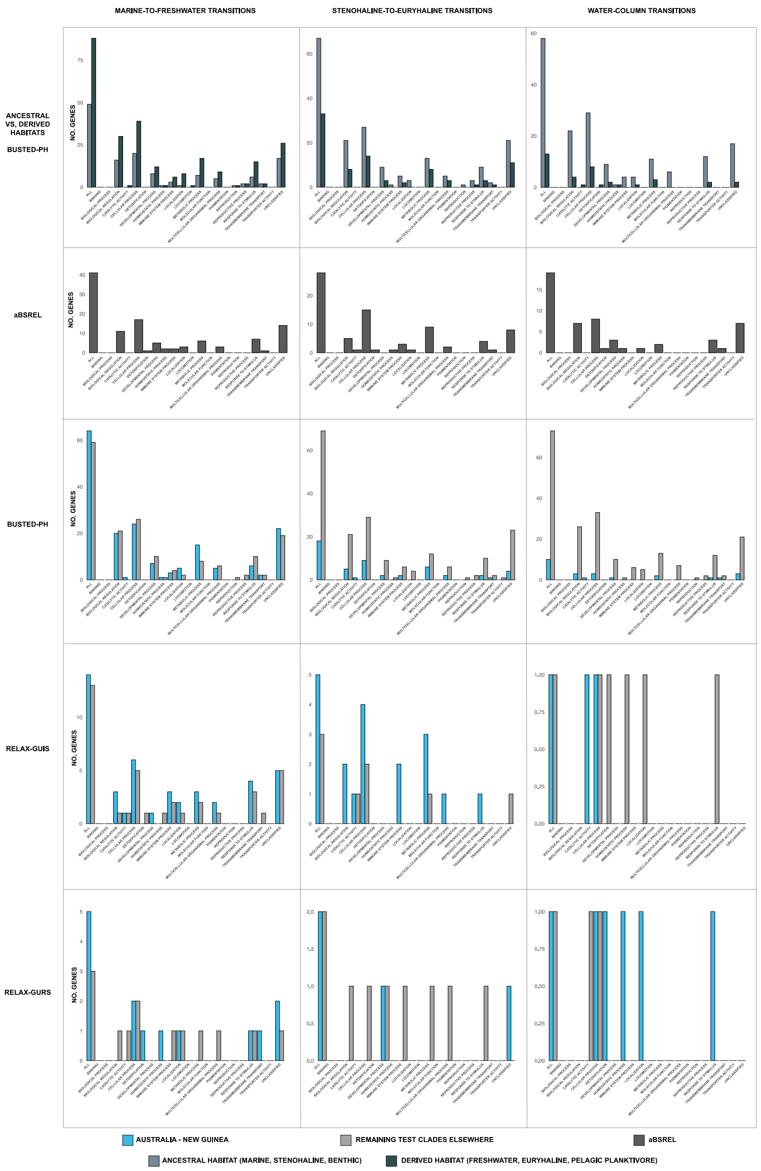

Figure S15. Genes under positive selection and changes in selection strength associated with twenty-one biological processes during the habitat transitions. For the ancestral against the derived habitats, a BUSTED-PH analysis was run, depicting the ancestral habitats in light slate gray and the derived ones in dark slate gray. In the aBSREL analysis, we focused on the stem lineage to identify genes under positive selection during the transition. In the BUSTED-PH and RELAX analyses, the cyan bars represent the AU-NG adaptive radiation, while the gray bars represent other derived clades worldwide. The RELAX analysis separates genes under intensification of selection (RELAX-GUIS) and relaxation of selection (RELAX-GURS) into separate panels.

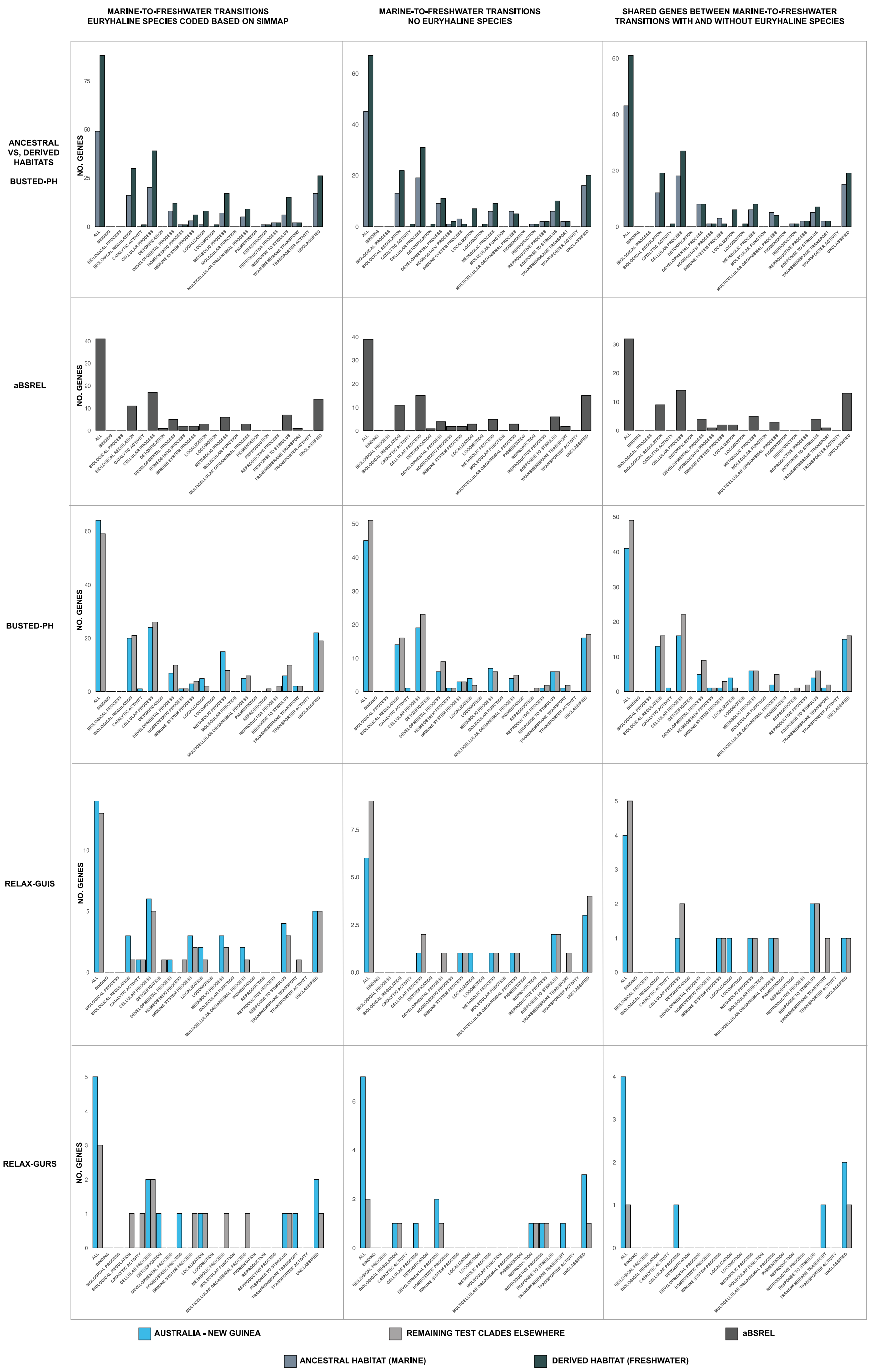

Figure S16. Genes under positive selection and changes in selection strength associated with twenty-one biological processes during the marine-to-freshwater transition. For the ancestral against the derived habitats, a BUSTED-PH analysis was run, depicting the ancestral habitats in light slate gray and the derived ones in dark slate gray. In the aBSREL analysis, we focused on the stem lineage to identify genes under positive selection during the transition. In the BUSTED-PH and RELAX analyses, the cyan bars represent the AU-NG adaptive radiation, while the gray bars represent other derived clades worldwide. The RELAX analysis separates genes under intensification of selection (RELAX-GUIS) and relaxation of selection (RELAX-GURS) into separate panels.

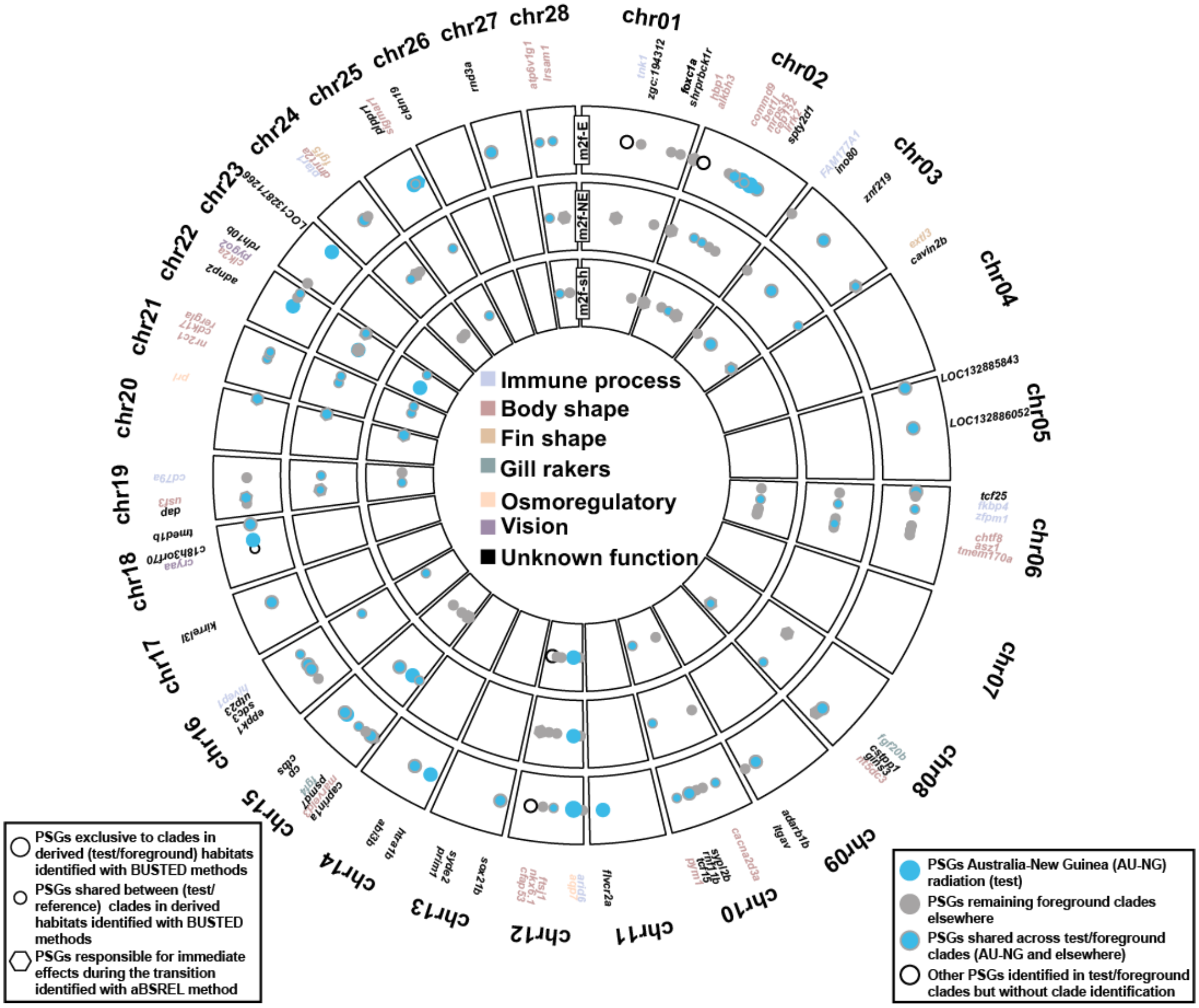

Figure S17. Location of positively selected genes (PSGs), identified by BUSTED-E, aBSREL, and BUSTED-PH, in association with marine-to-freshwater habitat transitions using the two coding schemes (m2f-E: marine-to-freshwater transitions including euryhaline species; m2f-NE: marine-to-freshwater transitions without euryhaline species), emphasizing the shared PSGs between the schemes (m2f-sh), relative to coordinates in the *Neoarius graeffei* genome, with color-coded functional categories. Each of the three-layered circles in the plot represents a habitat transition. m2f: marine-to-freshwater transitions; s2e: stenohaline-to-euryhaline transitions; b2p: benthic-to-pelagic planktivore transitions.

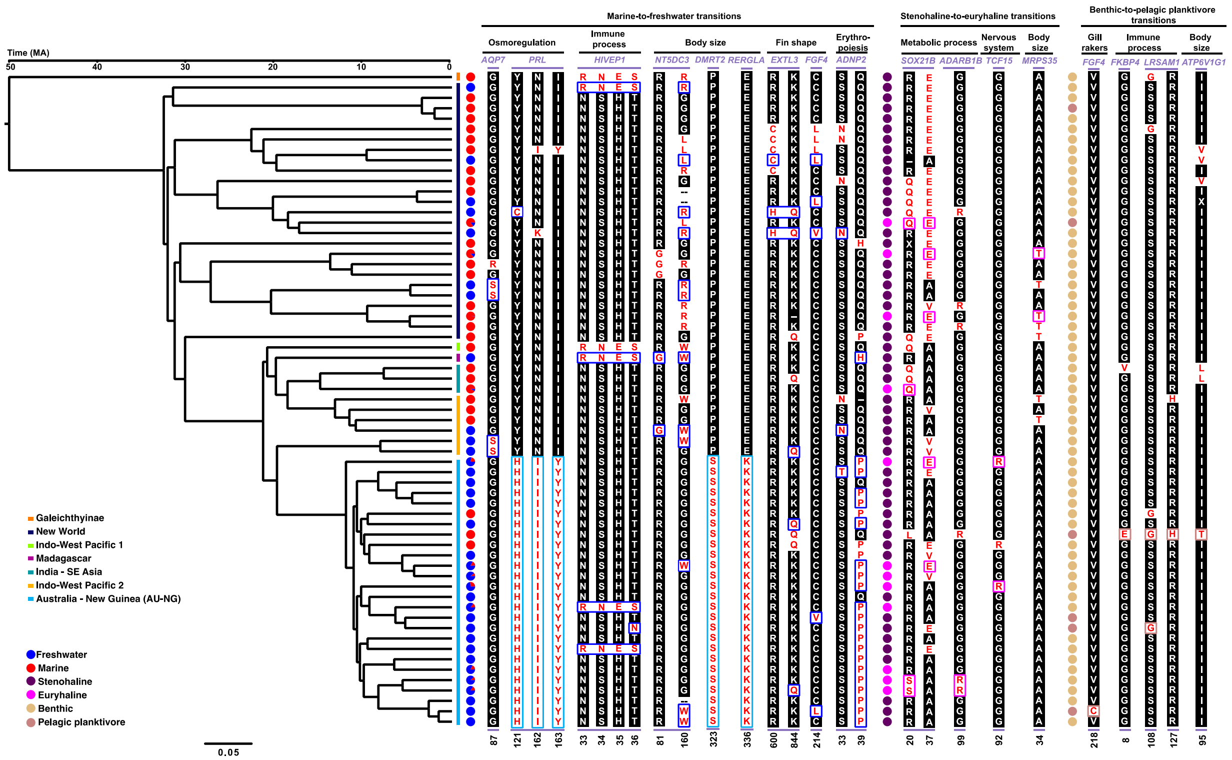

Figure S18. Alignment of PSS and amino acids. Marine-to-freshwater transitions: results of nine genes (*AQP7, PRL, HIVEP1, NT5DC3, DMRT2, RERGLA, EXTL3, FGF4, ADNP2*) with the lowest *p* values for branch-site results, related to specific functions linked to habitat transitions (e.g., osmoregulation, immune process, body size and elongation, fin shape, and erythropoiesis). Parallel sites under selection for MEME were: *AQP7*: 87; *PRL*: 163; *HIVEP1*: 33-36; *NT5DC3*: 81; *DMRT2*: 163; *RERGLA*: 336; *EXTL3*: 600, 844; *FGF4*: 214; *ADNP2*: 33. Convergent sites under selection for MEME were: *PRL*: 121, 162; *NT5DC3*: 160; *FGF4*: 214; *ADNP2*: 39. Stenohaline-to-euryhaline transitions: results of four genes (*SOX21B, ADARB1B, TCF15, MRPS35*) with the lowest *p* values for branch-site results, related to specific functions linked to habitat transitions (e.g., metabolic process, nervous system, and body size). Parallel sites under selection for MEME were: *SOX21B*: 37; *ADARB1B*: 99; *TCF15*: 92; *MRPS35*: 34. Convergent sites under selection for MEME were: *SOX21B*: 20. Benthic-to-pelagic planktivore transitions: results of four genes (*FGF4, FKBP4, LRSAM1, ATP6V1G1*) with the lowest p-values for branch-site results, related to specific functions linked to habitat transitions (e.g., gill rakers, immune process, body size). Parallel sites under selection for MEME were: *LRSAM1*: 108, 127. Convergent sites under selection for MEME were: *FKBP4*: 8; *ATP6V1G1*: 95. PSS in the derived habitats are shown in blue boxes for freshwater species, magenta for euryhaline species, and pink for pelagic planktivore species. PSS changes related to the adaptive radiation are shown in cyan boxes.

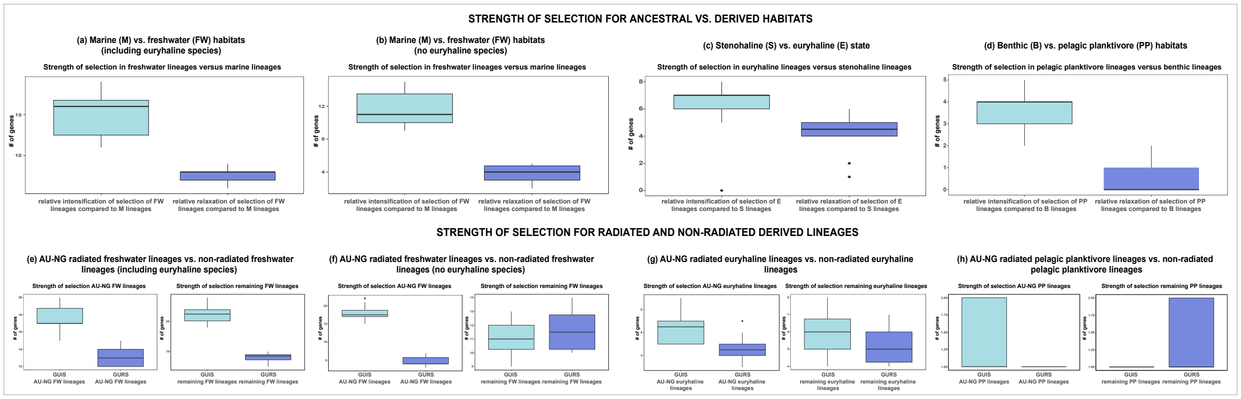

Figure S19. Strength of selection acting on genes during transitions to derived habitats across three distinct evolutionary radiations. Intensified selection is shown in green boxes, while relaxed selection is shown in purple boxes. The analyses reveal a prevailing trend of intensified selection in derived habitats across all transitions (a-d), observed in both radiating and non-radiating lineages (e-g). Freshwater lineages exhibited more genes under intensified selection (GUIS) than genes under relaxed selection (GURS) regardless of the presence of euryhaline species (a) or their exclusion (b). (c) The strength of selection in euryhaline lineages versus stenohaline lineages showed a higher number of genes under intensification of selection than relaxation of selection, without a significant difference in the number of genes. (d) Strength of selection in pelagic planktivore lineages versus benthic lineages showed a higher incidence of genes under intensification of selection, with relaxation of selection affecting at most one gene. Both radiating (AU-NG) and non-radiating freshwater lineages possessed a higher count of genes under intensified selection (e, f). However, the number of intensified genes was lower in non-radiating lineages when excluding euryhaline species (f). (g) Euryhaline lineages exhibited more genes under intensified selection than relaxed selection. Lastly, for (h) AU-NG radiating pelagic planktivore lineages vs. non-radiating pelagic planktivore a contrasting pattern emerged in the pelagic environment, with radiating and non-radiating lineages experiencing potentially opposing evolutionary forces, as evidenced by the contrasting selection patterns. The center line, box, and whiskers in the boxplots represent the median, interquartile range, and range of the data, respectively. Source data are provided as a Source Data file.

Morphological disparity and genes under selection for body shape and elongation. We conducted a reanalysis of the morphometric dataset for ariids[25], consisting of 28 morphometric (e.g., total length, standard length, eye diameter, interorbital distance) and 2 meristic (i.e., counts of gill rakers and anal fin rays) traits for 118 species. We implemented multivariate trait analysis to assess morphological disparity. Our aim was to investigate the impact of habitat (i.e., freshwater AU-NG adaptive radiation, freshwater in other regions, marine habitats) on the levels of morphological variation among ariids. To quantify morphological disparity across habitats, we calculated the sum of variances obtained from the first five principal component scores (~90% of the variance) based on the master tree. The results show that while freshwater species within the AU-NG adaptive radiation (average disparity= 224.2) exhibit slightly lower morphological disparity (224.2) compared to freshwater ariids found in other parts of the world (245.5), marine lineages show by far less variability in body size compared to their freshwater counterparts (103.1; Figure 4).

From a phylogenetic genotype-to-phenotype perspective, polygenic inheritance plays a crucial role in shaping body size and elongation in fishes[122]. As previously mentioned, a diverse set of genes contributes to various aspects of body shape, including elongation and overall body proportions. Thus, analyzing individual genes in isolation for body shape and elongation is not nearly as helpful at these macroevolutionary scales. However, the results of total morphological disparity can be compared directly with those of positive selection analyses. Of particular significance is the marked increase in body shape disparity in freshwater lineages overall (Figure 3; Figure S20), a finding that is line with the identification of a substantial number of genes (60-64) associated with body size and elongation under positive selection in these lineages (Figure S17). In the AU-NG adaptive radiation, we identified fewer genes under positive selection relative to other freshwater clades elsewhere, which is also consistent with a slightly lower total disparity in this clade (Figure 3; Figure S20). Notably, although a recent study did not find significant differences in body size disparity between marine and freshwater ariids[123], despite the fact that rates of evolution are indeed faster in freshwater species (see[25]), our analyses reveal that body shape disparity is higher in freshwater lineages compared to marine lineages (Figure 3; Figure S20; see also[25]).

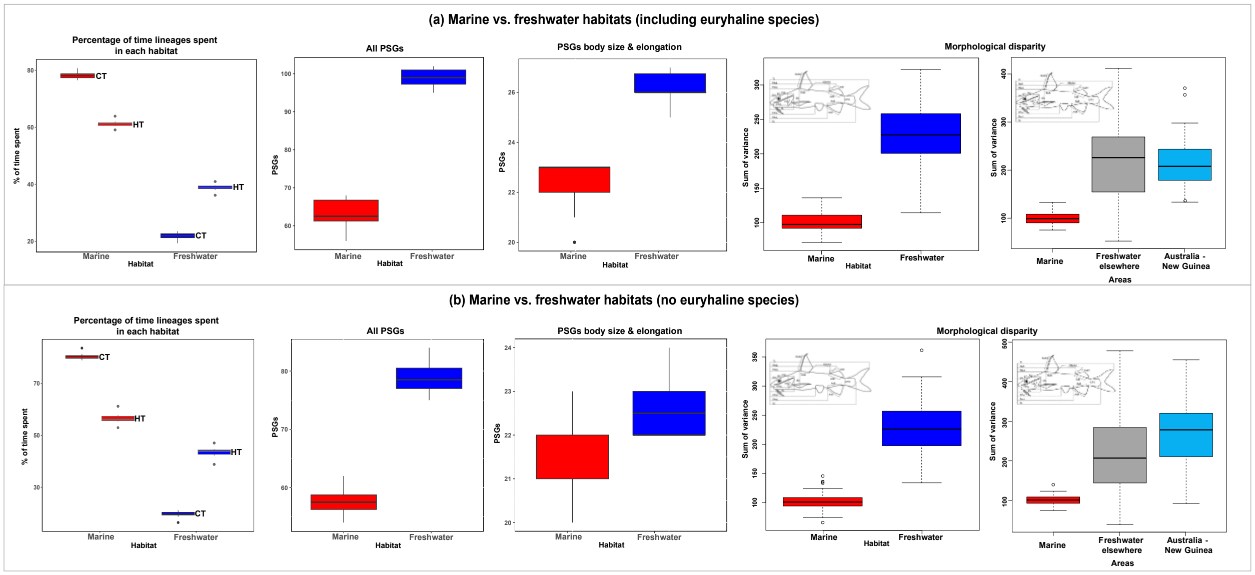

Figure S20. Evolutionary time spent by marine and freshwater ariid lineages in each habitat, along with the number of positively selected genes (PSGs) associated with body size and elongation, under two sampling strategies: (a) marine-to-freshwater transitions including euryhaline species, and (b) excluding euryhaline species. Time spent in each habitat is differentiated in two ways: (i) complete tree (CT; black stroke; 119 species), and (ii) sampling restricted to taxa with whole-genome sequences used for HyPhy analyses (HT; gray stroke; 66 species). In both scenarios, freshwater ariid lineages (blue) exhibit a higher number of PSGs compared to marine ariid lineages (red), aligning with the greater morphological disparity observed in the relatively younger freshwater ariid lineages. (a) Including euryhaline species: Marine ariid species spent approximately 78% (CT) and 61% (HT) of their evolutionary history in the ocean, while freshwater ariid species spent 22% (CT) and 39% (HT) in rivers. Mean disparity in freshwater ariid lineages was 234.85 (224.2 in AU-NG, 245.2 in non-radiating lineages), compared to 103.1 in marine lineages. (b) Excluding euryhaline species: Marine ariid species

spent approximately 80% (CT) and 56% (HT) of their evolutionary history in the ocean, while freshwater ariid species spent 20% (CT) and 44% (HT) in rivers. Mean disparity in freshwater ariid lineages was 269.3 (294.0 in AU-NG, 245.5 in non-radiating lineages), compared to 103.1 in marine ariid lineages. Boxplots depict the median (center line), interquartile range (box), and range (whiskers). Source data are provided as a Source Data file.

Supplementary Note 8

Repeat content & Transposable elements. To investigate whether genomic TE and repeat content varied across ariids and whether differences in specific TE and repeat families were correlated with ecological transitions, we used a phylogenetic ANOVA approach[124] (Figure S21). This testing was repeated to identify correlations in ten distinct TE and repeat classes (short interspersed nuclear elements (SINE), long interspersed nuclear elements (LINE), long terminal repeats (LTR), transposons (TRANSP), rolling circle elements (RC), unclassified repeats (UN), simple RNAs (SRNA), satellite elements (SAT), simple repeats (SR) and low complexity repeats (LC), across the 61 species dataset for each of the three transition axes (Supplementary Data S17a). To investigate whether genomic TE and repeat content varied across ariids and whether differences in specific TE and repeat families were correlated with ecological transitions, we used a phylogenetic ANOVA approach[124]. To account for multiple comparisons, we applied the p.adjust function in R to calculate the FDR and conducted the Benjamini-Hochberg procedure for p value correction. We considered results with a significance threshold of 0.05 as statistically significant (Supplementary Data S17b). Among ariid species, retroelements emerged as the dominant category within repeat classes, followed closely by DNA transposons (Figure S21; Supplementary Data S17a). Additionally, marine, euryhaline, and pelagic planktivore species exhibited a greater proportion of the genome covered by TE and repeat classes in comparison to their freshwater, stenohaline, and benthic counterparts (Figure 4; Figure S21). Despite notable differences observed among the groups, our analysis did not reveal significant associations (FDR corrected p values < 0.05) between the content of TE and repeat families and habitat across all three axes of variation.

In addition to the accumulation of adaptive variation in genic regions that underpinning facilitates adaptation to new habitats, changes to other aspects of the genome including TEs and repeat content have also been shown to drive, and result from, adaptation to new environments[125]. However, due to the variation in the distribution and function of TEs and repeats across different fish species and the general complexity of the genomes of many fish species[126] it can be challenging to confidently identify TEs across the genome and to link TEs and repeat diversity to evolutionary patterns and processes across clades. The copy number and composition of TEs vary substantially among different fish taxa, with percentages ranging from approximately 6% in the green spotted pufferfish to around 55% in the zebrafish[126,127]. Remarkably, the lesser salmon catfish, with a genome size of approximately 2.34 Gbp, exhibits a high abundance of TEs, constituting a total interspersed repeat content of 69.23%. The fish genome of the lesser salmon catfish is

predominantly composed of LTR and LINE elements, while SINEs are generally scarce, consistent with observations in other fish species[127]. Although adaptation to new environments has been shown to be accompanied by dramatic changes in repeat and TE content[128], we did not detect any significant consistent changes in repeat families across the ariid clade. This result may indicate that substantial adaptive changes in response to habitat transitions may first evolve as a result of selection directly on mutations in coding genic regions. It also raises the possibilities that the habitat transitions we investigate here occurred too gradually, were not immediately accompanied by complete reproductive isolation, or occurred too recently meaning that independent changes in repeat content may have not accumulated in different lineages.

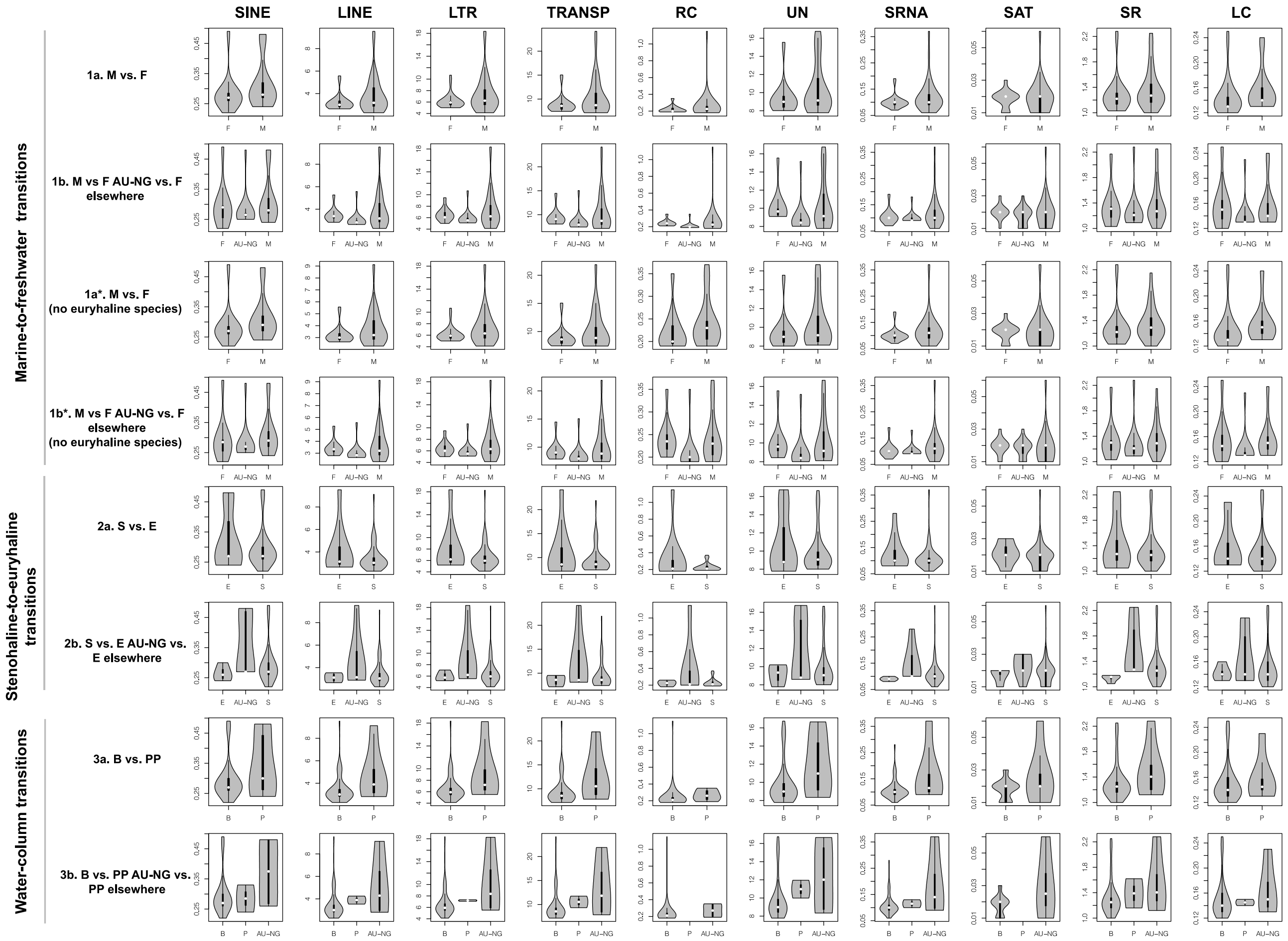

Figure S21. Violin plots highlighting differences in the proportion of the genome that different TE and repeat families span. Genome proportions for each TE and repeat class across the 61 species were plotted including short interspersed nuclear elements (SINE), long interspersed nuclear elements (LINE), long terminal repeats (LTR), transposons (TRANSP), rolling circle elements (RC), unclassified repeats (UN), simple RNAs (SRNA), satellite elements (SAT), simple repeats (SR) and low complexity repeats (LC), with individuals grouped by each ecological contrast that was tested. These include 1a) marine vs. freshwater (euryhaline species codified considered the most likely binary tip state as inferred with SIMMAP), 1a*) marine vs. freshwater (no euryhaline species), 1b) marine vs. freshwater AU-NG species vs. all other freshwater species (euryhaline species codified considered the most likely binary tip state as

inferred with SIMMAP), 1b*) marine vs. freshwater AU-NG species vs. all other freshwater species (no euryhaline species) 2a) stenohaline vs. euryhaline, 2b) stenohaline vs. euryhaline species from AU-NG vs. all other euryhaline species, 3a) benthic vs. pelagic planktivore , and 3b) benthic vs. pelagic planktivorous species from AU-NG, vs. all other pelagic planktivore species. TE and repeat contents with significant phylogenetic ANOVA values (FDR corrected p values < 0.05) are plotted in color while non-significant tests are in gray.

References

1. Arcila, D. *et al.* Genome-wide interrogation advances resolution of recalcitrant groups in the tree of life. *Nat. Ecol. Evol.* (2017). doi:10.1038/s41559-016-0020
2. Larivière, D. *et al.* Scalable, accessible and reproducible reference genome assembly and evaluation in Galaxy. *Nat. Biotechnol.* **42**, 367–370 (2024).
3. Cheng, H., Concepcion, G. T., Feng, X., Zhang, H. & Li, H. Haplotype-resolved de novo assembly using phased assembly graphs with hifiasm. *Nat. Methods* **18**, 170–175 (2021).
4. Gurevich, A., Saveliev, V., Vyahhi, N. & Tesler, G. QUAST: Quality assessment tool for genome assemblies. *Bioinformatics* **29**, 1072–1075 (2013).
5. Simão, F. A., Waterhouse, R. M., Ioannidis, P., Kriventseva, E. V. & Zdobnov, E. M. BUSCO: Assessing genome assembly and annotation completeness with single-copy orthologs. *Bioinformatics* **31**, 3210–3212 (2015).
6. Manni, M., Berkeley, M. R., Seppey, M., Simão, F. A. & Zdobnov, E. M. BUSCO Update: Novel and Streamlined Workflows along with Broader and Deeper Phylogenetic Coverage for Scoring of Eukaryotic, Prokaryotic, and Viral Genomes. *Mol. Biol. Evol.* **38**, 4647–4654 (2021).
7. Rhie, A., Walenz, B. P., Koren, S. & Phillippy, A. M. Merqury: Reference-free quality, completeness, and phasing assessment for genome assemblies. *Genome Biol.* **21**, 1–27 (2020).
8. Harry, E. PretextView (Paired REad TEXTure Viewer): A desktop application for viewing pretext contact maps. (2023).
9. Luo, R. *et al.* SOAPdenovo2: An empirically improved memory-efficient short-read de novo assembler. *Gigascience* **1**, 6 (2012).
10. Andrews, S. FastQC: A Quality Control Tool for High Throughput Sequence Data. (2010).
11. Bolger, A. M., Lohse, M. & Usadel, B. Trimmomatic: a flexible trimmer for Illumina sequence data. *Bioinformatics* **30**, 2214–2120 (2014).
12. Bankevich, A. *et al.* SPAdes: A new genome assembly algorithm and its applications to single-cell sequencing. *J. Comput. Biol.* **19**, 455–477 (2012).
13. Alonge, M. *et al.* RaGOO: Fast and accurate reference-guided scaffolding of draft genomes. *Genome Biol.* **20**, 1–17 (2019).
14. Li, H. & Durbin, R. Fast and accurate short read alignment with Burrows – Wheeler transform. *Bioinformatics* **25**, 1754–1760 (2009).
15. Li, H. *et al.* The Sequence Alignment/Map format and SAMtools. *Bioinformatics* **25**, 2078–2079 (2009).
16. Zerbino, D. R. & Birney, E. Velvet: Algorithms for de novo short read assembly using de Bruijn graphs. *Genome Res.* **18**, 821–829 (2008).
17. Allen, J. M., LaFrance, R., Folk, R. A., Johnson, K. P. & Guralnick, R. P. aTRAM 2.0: An Improved, Flexible Locus Assembler for NGS Data. *Evol. Bioinforma.* **14**, 0–3 (2018).
18. Fu, L., Niu, B., Zhu, Z., Wu, S. & Li, W. CD-HIT: Accelerated for clustering the next-generation sequencing data. *Bioinformatics* **28**, 3150–3152 (2012).
19. Slater, G. S. C. & Birney, E. Automated generation of heuristics for biological sequence comparison. *BMC Bioinformatics* **6**, 1–11 (2005).
20. Ranwez, V., Harispe, S., Delsuc, F. & Douzery, E. J. P. MACSE: Multiple alignment of coding SEquences accounting for frameshifts and stop codons. *PLoS One* **6**, (2011).
21. Wheeler, T. J. & Eddy, S. R. Nhmmer: DNA homology search with profile HMMs. *Bioinformatics* **29**, 2487–2489 (2013).

22. Kearse, M. *et al.* Geneious Basic: An integrated and extendable desktop software platform for the organization and analysis of sequence data. *Bioinformatics* **28**, 1647–1649 (2012).
23. Betancur-R., R., Acero P., A., Bermingham, E. & Cooke, R. Systematics and biogeography of New World sea catfishes (Siluriformes: Ariidae) as inferred from mitochondrial, nuclear, and morphological evidence. *Mol. Phylogenet. Evol.* **45**, 339–357 (2007).
24. Betancur-R, R. Molecular phylogenetics supports multiple evolutionary transitions from marine to freshwater habitats in ariid catfishes. *Mol. Phylogenet. Evol.* **55**, 249–258 (2010).
25. Betancur R., R., Ortí, G., Stein, A. M., Marceniuk, A. P. & Alexander Pyron, R. Apparent signal of competition limiting diversification after ecological transitions from marine to freshwater habitats. *Ecol. Lett.* **15**, 822–830 (2012).
26. Revell, L. J. phytools: An R package for phylogenetic comparative biology (and other things). *Methods Ecol. Evol.* **3**, 217–223 (2012).
27. Rincon-Sandoval, M. *et al.* Evolutionary determinism and convergence associated with water-column transitions in marine fishes. *PNAS* **117**, 33396–33403 (2020).
28. Santaquiteria, A. *et al.* Evolutionary history and biogeography of seahorses, dragonets, goatfishes and allies (Teleostei: Syngnatharia). *Syst. Biol.* **0**, 1–18 (2021).
29. Peterson, R. D. *et al.* Phylogenomics of Bony-Tongue Fishes (Osteoglossomorpha) Shed Light on the Craniofacial Evolution and Biogeography of the Weakly Electric Clade (Mormyridae). *Syst. Biol.* **71**, 1032–1044 (2022).
30. Mendes, F. K. & Hahn, M. W. Gene tree discordance causes apparent substitution rate variation. *Syst. Biol.* **65**, 711–721 (2016).
31. Lanfear, R., Frandsen, P. B., Wright, A. M., Senfeld, T. & Calcott, B. Partitionfinder 2: New methods for selecting partitioned models of evolution for molecular and morphological phylogenetic analyses. *Mol. Biol. Evol.* **34**, 772–773 (2017).
32. Kozlov, A. M., Darriba, D., Flouri, T., Morel, B. & Stamatakis, A. RAxML-NG: A fast, scalable and user-friendly tool for maximum likelihood phylogenetic inference. *Bioinformatics* **35**, 4453–4455 (2019).
33. Zhang, C., Rabiee, M., Sayyari, E. & Mirarab, S. ASTRAL-III: Polynomial time species tree reconstruction from partially resolved gene trees. *BMC Bioinformatics* **19**, 15–30 (2018).
34. Emms, D. M. & Kelly, S. OrthoFinder: Phylogenetic orthology inference for comparative genomics. *bioRxiv* 1–14 (2018). doi:10.1101/466201
35. Emms, D. M. & Kelly, S. STAG: Species Tree Inference from All Genes. *bioRxiv* 1–29 (2018). doi:https://doi.org/10.1101/267914
36. Yang, Z. PAML 4: Phylogenetic Analysis by Maximum Likelihood. *Mol. Biol. Evol.* **24**, 1586–1591 (2007).
37. dos Reis, M. & Yang, Z. Bayesian Molecular Clock Dating Using Genome-Scale Datasets. in *Evolutionary Genomics: Statistical and Computational Methods* (Springer Protocols, 2019).
38. Stange, M., Sánchez-Villagra, M. R., Salzburger, W. & Matschiner, M. Bayesian divergence-time estimation with genome-wide single-nucleotide polymorphism data of sea catfishes (Ariidae) supports miocene closure of the Panamanian Isthmus. *Syst. Biol.* **67**, 681–699 (2018).
39. Sabaj Pérez, M. H., Aguilera S, O. A. & Lundberg, J. G. Fossil Catfishes of the Families Doradidae and Pimelodidae (Teleostei: Siluriformes) from the Miocene Urumaco Formation of Venezuela. *Proc. Acad. Nat. Sci. Philadelphia* **156**, 157–194 (2007).
40. Robertson, D. R. & Allen, G.-R. *Shorefishes of the Tropical Eastern Pacific: online information system.* (2015).
41. Robertson, D. R. & Van Tassell, J. *Shorefishes of the Greater Caribbean: online information system*. (2016).
42. Kailola, P. J. A phylogenetic exploration of the catfish family Ariidae (Otophysi : Siluriformes). *Beagle, Rec. Museums Art Gall. North. Territ.* **20**, 79–156 (2004).
43. Allen, G. R., Midgley, S. H. & Allen, M. *Field guide to the freshwater fishes of Australia*. (CSIRO Publisher, 2002).

44. Allen, G. R. *Field guide to the freshwater fishes of New Guinea*. (Christensen Research Institute, 1991).
45. Bollback, J. P. SIMMAP: Stochastic character mapping of discrete traits on phylogenies. *BMC Bioinformatics* **7**, (2006).
46. Lundsgaard-Hansen, B., Matthews, B., Vonlanthen, P., Taverna, A. & Seehausen, O. Adaptive plasticity and genetic divergence in feeding efficiency during parallel adaptive radiation of whitefish (Coregonus spp.). *J. Evol. Biol.* **26**, 483–498 (2013).
47. Glazer, A. M., Cleves, P. A., Erickson, P. A., Lam, A. Y. & Miller, C. T. Parallel developmental genetic features underlie stickleback gill raker evolution. *Evodevo* **5**, 1–16 (2014).
48. Malinsky, M. *et al.* Whole-genome sequences of Malawi cichlids reveal multiple radiations interconnected by gene flow. *Nat. Ecol. Evol.* **2**, 1940–1955 (2018).
49. Marques, D. A., Jones, F. C., Di Palma, F., Kingsley, D. M. & Reimchen, T. E. Experimental evidence for rapid genomic adaptation to a new niche in an adaptive radiation. *Nat. Ecol. Evol.* **2**, (2018).
50. De-Kayne, R. *et al.* Genomic architecture of adaptive radiation and hybridization in Alpine whitefish. *Nat. Commun.* **13**, 1–13 (2022).
51. Delorenzo, L. *et al.* Genetic basis of ecologically relevant body shape variation among four genera of cichlid fishes. *Mol. Ecol.* 1–14 (2023). doi:10.1111/mec.16977
52. Bogan, S. & Fernández, E. Presencia del bagre marino Genidens planifrons (Teleostei, Siluriformes, Ariidae) en las costas de la República Argentina. *Rev. del Mus. Argentino Ciencias Nat.* **15**, 107–111 (2013).
53. Kailola, P. J. Six New Species of Fork-tailed Catfishes (Pisces, Teleostei, Ariidae) from Australia and New Guinea. *Beagle Rec. Museums Art Gall. North. Territ.* **16**, 127–144 (2000).
54. Cooke, R. & Jiménez, M. Teasing out the species in diverse archaeofaunas: Is it worth the effort? An example from the tropical eastern pacific. *Archaeofauna* **13**, 19–35 (2004).
55. Froese, R. & Pauly, D. FishBase. (2019). Available at: www.fishbase.org.
56. Fabrício Lemos de Siqueira Mendes & Ronaldo Borges Barthem. Hábitos alimentares de bagres marinhos (Siluriformes: Ariidae) do estuário amazônico. *Amaz. Ciência Desenvolv.* **5**, 153–166 (2010).
57. Pusey, B. J., Jardine, T. D., Bunn, S. E. & Douglas, M. M. Sea catfishes (Ariidae) feeding on freshwater floodplains of northern Australia. *Mar. Freshw. Res.* **71**, 1628–1639 (2020).
58. Pond, S. L. K. *et al.* HyPhy 2 . 5 — A Customizable Platform for Evolutionary Hypothesis Testing Using Phylogenies. *Mol. Biol. Evol.* **37**, 295–299 (2019).
59. Glor, R. E. Phylogenetic insights on adaptive radiation. *Annu. Rev. Ecol. Evol. Syst.* **41**, 251–270 (2010).
60. Murrell, B. *et al.* Gene-wide identification of episodic selection. *Mol. Biol. Evol.* **32**, 1365–1371 (2015).
61. Wisotsky, S. R., Pond, S. L. K., Shank, S. D. & Muse, S. V. Synonymous site-to-site substitution rate variation dramatically inflates false positive rates of selection analyses: Ignore at your own peril. *Mol. Biol. Evol.* **37**, 2430–2439 (2021).
62. Smith, M. D. *et al.* Less is more: An adaptive branch-site random effects model for efficient detection of episodic diversifying selection. *Mol. Biol. Evol.* **32**, 1342–1353 (2015).
63. Wertheim, J. O., Murrell, B., Smith, M. D., Pond, S. L. K. & Scheffler, K. RELAX: Detecting relaxed selection in a phylogenetic framework. *Mol. Biol. Evol.* **32**, 820–832 (2015).
64. Murrell, B. *et al.* Detecting individual sites subject to episodic diversifying selection. *PLoS Genet.* **8**, (2012).
65. Guillerme, T. dispRity : A modular R package for measuring disparity. *Methods Ecol. Evol.* 1–9 (2018). doi:10.1111/2041-210X.13022
66. Seppey, M., Manni, M. & Zdobnov, E. M. BUSCO: Assessing Genome Assembly and Annotation Completeness. *Methods Mol. Biol.* **1962**, (2019).
67. Wang, H., Su, B., Butts, I. A. E., Dunham, R. A. & Wang, X. Chromosome-level assembly and

annotation of the blue catfish Ictalurus furcatus, an aquaculture species for hybrid catfish reproduction, epigenetics, and heterosis studies. *Gigascience* **11**, 1–19 (2022).
68. Marceniuk, A. P., Oliveira, C. & Ferraris Jr, C. J. A new classification of the family Ariidae (Osteichthyes : Ostariophysi : Siluriformes) based on combined analyses of morphological and molecular data. *Zool. J. Linn. Soc.* **XX**, 1–51 (2023).
69. Acero, A. & Betancur-R, R. *Real identity of the northern Colombian endemic sea catfish Galeichthys bonillai Miles, 1945 (Siluriformes: Ariidae)*. *Cybium* **30**, (2006).
70. Acero P., A. & Betancur-R., R. Monophyly, affinities, and subfamilial clades of sea catfishes (Siluriformes: Ariidae). *Ichthyol. Explor. Freshwaters* **18**, 133–143 (2007).
71. Ahi, E. P., Richter, F., Lecaudey, L. A. & Sefc, K. M. Gene expression profiling suggests differences in molecular mechanisms of fin elongation between cichlid species. *Sci. Rep.* **9**, 1–13 (2019).
72. Ahi, E. P. & Sefc, K. M. Towards a gene regulatory network shaping the fins of the Princess cichlid. *Sci. Rep.* **8**, 1–13 (2018).
73. Velotta, J. P. *et al.* Transcriptomic imprints of adaptation to fresh water: parallel evolution of osmoregulatory gene expression in the Alewife. *Mol. Ecol.* **26**, 831–848 (2017).
74. Velotta, J. P., McCormick, D. & Jones, A. W. Reduced Swimming Performance Repeatedly Evolves on Loss of Migration in Landlocked Populations of Alewife. *Physiol. Biochem. Zool.* **91**, 814–825 (2018).
75. Ishikawa, A. *et al.* A key metabolic gene for recurrent freshwater colonization and radiation in fishes. *Science (80-.).* **364**, 886–889 (2019).
76. Roycroft, E. *et al.* Molecular Evolution of Ecological Specialisation: Genomic Insights from the Diversification of Murine Rodents. *Genome Biol. Evol.* **13**, 1–16 (2021).
77. Smith, S. D., Pennell, M. W., Dunn, C. W. & Edwards, S. V. Phylogenetics is the New Genetics (for Most of Biodiversity). *Trends Ecol. Evol.* **35**, 415–425 (2020).
78. Dalziel, A. C., Rogers, S. M. & Schulte, P. M. Linking genotypes to phenotypes and fitness: How mechanistic biology can inform molecular ecology. *Mol. Ecol.* **18**, 4997–5017 (2009).
79. de Brito, V. *et al.* Patterns of Phenotypic Evolution Associated with Marine/Freshwater Transitions in Fishes. *Integr. Comp. Biol.* **62**, 406–423 (2022).
80. Ahi, E. P., Richter, F. & Sefc, K. M. A gene expression study of ornamental fin shape in Neolamprologus brichardi, an African cichlid species. *Sci. Rep.* **7**, 1–14 (2017).
81. Suzzi, S. *et al.* Deletion of lrrk2 causes early developmental abnormalities and age-dependent increase of monoamine catabolism in the zebrafish brain. *PLoS Genet.* **17**, 1–30 (2021).
82. Willekers, S. *et al.* The centriolar satellite protein Cfap53 facilitates formation of the zygotic microtubule organizing center in the zebrafish embryo. *Dev.* **149**, (2022).
83. Wang, J. *et al.* Methylation of HBP1 by PRMT1 promotes tumor progression by regulating actin cytoskeleton remodeling. *Oncogenesis* **11**, (2022).
84. Holmes, G., Ferguson, S. R., Lewis, P. A. & Echeverri, K. LRRK2 kinase activity is necessary for development and regeneration in Nematostella vectensis. 1–21 (2023).
85. Tort, L. Stress and immune modulation in fish. *Dev. Comp. Immunol.* **35**, 1366–1375 (2011).
86. Franke, A., Beemelmanns, A. & Miest, J. J. Are fish immunocompetent enough to face climate change? *Biol. Lett.* **20**, (2024).
87. Liu, Y. *et al.* Grass carp (Ctenopharyngodon idella) TNK1 modulates JAK-STAT signaling through phosphorylating STAT1. *Dev. Comp. Immunol.* **116**, 103951 (2021).
88. Gouife, M. *et al.* Identification and functional characterization of Interleukin-11 in goldfish (Carassius auratus L.). *Fish Shellfish Immunol. Reports* **5**, 0–9 (2023).
89. Auffret, P. *et al.* Transgenerational exposure to ocean acidification impacts the hepatic transcriptome of European sea bass (Dicentrarchus labrax). *BMC Genomics* **24**, 1–11 (2023).
90. Yan, W. *et al.* The hsp40 Gene Family in Japanese Flounder: Identification, Phylogenetic Relationships, Molecular Evolution Analysis, and Expression Patterns. *Front. Mar. Sci.* **7**, 1–16 (2021).

91. Taugbøl, A., Solbakken, M. H., Jakobsen, K. S. & Vøllestad, L. A. Salinity-induced transcriptome profiles in marine and freshwater threespine stickleback after an abrupt 6-hour exposure. *Ecol. Evol.* **12**, 1–15 (2022).
92. Cvekl, A. & Ashery-Padan, R. The cellular and molecular mechanisms of vertebrate lens development. *Dev.* **141**, 4432–4447 (2014).
93. Ahi, E. P., Richter, F. & Sefc, K. M. Gene expression patterns associated with caudal fin shape in the cichlid Lamprologus tigripictilis. *Hydrobiologia* (2022). doi:10.1007/s10750-022-05068-4
94. Volpi, S. *et al.* EXTL3 mutations cause skeletal dysplasia, immune deficiency, and developmental delay. *J. Exp. Med.* **214**, 623–637 (2017).
95. Notarangelo, L. D. Expanding the spectrum of skeletal dysplasia with immunodeficiency: A commentary on identification of biallelic EXTL3 mutations in a novel type of spondylo-epi-metaphyseal dysplasia. *J. Hum. Genet.* **62**, 737–738 (2017).
96. Oliver, T. A. *et al.* Whole-genome positive selection and habitat-driven evolution in a shallow and a deep-sea urchin. *Genome Biol. Evol.* **2**, 800–814 (2010).
97. Vogt, G. Environmental Adaptation of Genetically Uniform Organisms with the Help of Epigenetic Mechanisms—An Insightful Perspective on Ecoepigenetics. *Epigenomes* **7**, (2023).
98. Lu, B., Jin, H. & Fu, J. Molecular convergent and parallel evolution among four high-elevation anuran species from the Tibetan region. *BMC Genomics* **21**, 1–14 (2020).
99. Eastment, R. V., Wong, B. B. M. & McGee, M. D. Convergent genomic signatures associated with vertebrate viviparity. *BMC Biol.* **22**, 34 (2024).
100. Yang, A. *et al.* An essential role of high-molecular-weight kininogen in endotoxemia. *J. Exp. Med.* **214**, 2649–2670 (2017).
101. Das, S. & Sahoo, P. K. Ceruloplasmin, a moonlighting protein in fish. *Fish Shellfish Immunol.* **82**, 460–468 (2018).
102. Abt, E. R. *et al.* Purine nucleoside phosphorylase enables dual metabolic checkpoints that prevent T cell immunodeficiency and TLR7-associated autoimmunity. *J. Clin. Invest.* **132**, 1–15 (2022).
103. Varga, M. *et al.* Tissue-Specific Requirement for the GINS Complex During Zebrafish Development. *Front. Cell Dev. Biol.* **8**, 1–13 (2020).
104. Arokiasamy, S., Balderstone, M. J. M., De Rossi, G. & Whiteford, J. R. Syndecan-3 in Inflammation and Angiogenesis. *Front. Immunol.* **10**, 1–7 (2020).
105. Wang, J. *et al.* Resolving the genetic paradox of invasions: Preadapted genomes and postintroduction hybridization of bigheaded carps in the Mississippi River Basin. *Evol. Appl.* **13**, 263–277 (2020).
106. Storz, J. F. Causes of molecular convergence and parallelism in protein evolution. *Nat. Rev. Genet.* **17**, 239–250 (2017).
107. He, X. *et al.* The Zinc Transporter SLC39A10 Plays an Essential Role in Embryonic Hematopoiesis. *Adv. Sci.* **10**, 1–17 (2023).
108. InterPro. DNA primase small subunit. Available at: https://www.ebi.ac.uk/interpro/protein/UniProt/P49642/. (Accessed: 4th November 2024)
109. Aristide, L. & Fernández, R. Genomic Insights into Mollusk Terrestrialization: Parallel and Convergent Gene Family Expansions as Key Facilitators in Out-of-the-Sea Transitions. *Genome Biol. Evol.* **15**, 1–17 (2023).
110. Tine, M. *et al.* European sea bass genome and its variation provide insights into adaptation to euryhalinity and speciation. *Nat. Commun.* **5**, 5770 (2014).
111. Takvam, M., Wood, C. M., Kryvi, H. & Nilsen, T. O. Ion Transporters and Osmoregulation in the Kidney of Teleost Fishes as a Function of Salinityfile:///Users/melissarincon/The Whisky Xperience Dropbox/Melissa Rincon/OU_POSTDOC/ARIIDAE_PAPER/PAPERS_GENOMICS/2018.Weaver.pdf. *Front. Physiol.* **12**, 1–25 (2021).
112. Kolosov, D., Bui, P., Chasiotis, H. & Kelly, S. P. Claudins in teleost fishes. *Tissue Barriers* **1**, e25391 (2013).

113. Sackton, T. B. & Clark, N. Convergent evolution in the genomics era: New insights and directions. *Philos. Trans. R. Soc. B Biol. Sci.* **374**, 24–27 (2019).
114. Van Eeden, F. J. M. *et al.* Genetic analysis of fin formation in the zebrafish, Danio rerio. *Development* **123**, 255–262 (1996).
115. Gillis, J. A., Dahn, R. D. & Shubin, N. H. Shared developmental mechanisms pattern the vertebrate gill arch and paired fin skeletons. *Proc. Natl. Acad. Sci. U. S. A.* **106**, 5720–5724 (2009).
116. Cooper, W. J., Wirgau, R. M., Sweet, E. M. & Albertson, R. C. Deficiency of zebrafish fgf20a results in aberrant skull remodeling that mimics both human cranial disease and evolutionarily important fish skull morphologies. *Evol Dev* **15**, 426–441 (2013).
117. Leck, K. J., Zhang, S. & Hauser, C. A. E. Study of bioengineered zebra fish olfactory receptor 131-2: Receptor purification and secondary structure analysis. *PLoS One* **5**, (2010).
118. Li, X. C., Wang, X. W., Wang, Z. H., Zhao, X. F. & Wang, J. X. A three-domain Kazal-type serine proteinase inhibitor exhibiting domain inhibitory and bacteriostatic activities from freshwater crayfish Procambarus clarkii. *Dev. Comp. Immunol.* **33**, 1229–1238 (2009).
119. Hooda, J., Shah, A. & Zhang, L. Heme, an essential nutrient from dietary proteins, critically impacts diverse physiological and pathological processes. *Nutrients* **6**, 1080–1102 (2014).
120. Dong, J. *et al.* Comparative Genomics Studies on the dmrt Gene Family in Fish. *Front. Genet.* **11**, 1–17 (2020).
121. Lachance, V. *et al.* Overview of Sigma-1R Subcellular Specific Biological Functions and Role in Neuroprotection. *Int. J. Mol. Sci.* **24**, (2023).
122. Zhang, W. *et al.* The genetic architecture of phenotypic diversity in the Betta fish (Betta splendens). *Sci. Adv.* **8**, (2022).
123. de Brito, V. *et al.* Species interactions and niche conservatism limit phenotypic diversification following marine/freshwater transitions in fishes. in *The Society for Integrative and Comparative Biology* Phoeniz, AZ (2022).
124. Collyer, M. L. & Adams, D. C. RRPP: Linear Model Evaluation with Randomized Residuals in a Permutation Procedure. (2019).
125. Casacuberta, E. & González, J. The impact of transposable elements in environmental adaptation. *Mol. Ecol.* **22**, 1503–1517 (2013).
126. Sotero-Caio, C. G., Platt, R. N., Suh, A. & Ray, D. A. Evolution and diversity of transposable elements in vertebrate genomes. *Genome Biol. Evol.* **9**, 161–177 (2017).
127. Shao, F., Han, M. & Peng, Z. Evolution and diversity of transposable elements in fish genomes. *Sci. Rep.* **9**, 1–8 (2019).
128. Hancock, Z. B., Hardin, F. O., Murthy, A., Hillhouse, A. & Johnston, J. S. Rapid genomic expansion and purging associated with habitat transitions in a clade of beach crustaceans (Amphipoda: Haustoriidae). *J. Crustac. Biol.* **41**, 1–11 (2021).
