# Supplementary material for: Ecological diversification of sea catfishes is accompanied by genome-wide signatures of positive selection
Source: Nat Commun. 2024 Nov 20;15:10040. doi: 10.1038/s41467-024-54184-3 (PMC11579386; doi:10.1038/s41467-024-54184-3)
Supplement: Supplementary file 2 — Reporting Summary [file 41467_2024_54184_MOESM2_ESM.pdf]

nature portfolio

Corresponding author(s): Ricardo Betancur-R

Last updated by author(s): 2024/10/01

Reporting Summary

Nature Portfolio wishes to improve the reproducibility of the work that we publish. This form provides structure for consistency and transparency in reporting. For further information on Nature Portfolio policies, see our Editorial Policies and the Editorial Policy Checklist.

Statistics

For all statistical analyses, confirm that the following items are present in the figure legend, table legend, main text, or Methods section.

n/a	Confirmed	
☐	☒	The exact sample size (*n*) for each experimental group/condition, given as a discrete number and unit of measurement
☐	☒	A statement on whether measurements were taken from distinct samples or whether the same sample was measured repeatedly
☒	☐	The statistical test(s) used AND whether they are one- or two-sided<br>*Only common tests should be described solely by name; describe more complex techniques in the Methods section.*
☒	☐	A description of all covariates tested
☐	☒	A description of any assumptions or corrections, such as tests of normality and adjustment for multiple comparisons
☐	☒	A full description of the statistical parameters including central tendency (e.g. means) or other basic estimates (e.g. regression coefficient) AND variation (e.g. standard deviation) or associated estimates of uncertainty (e.g. confidence intervals)
☐	☒	For null hypothesis testing, the test statistic (e.g. *F*, *t*, *r*) with confidence intervals, effect sizes, degrees of freedom and *P* value noted<br>*Give P values as exact values whenever suitable.*
☐	☒	For Bayesian analysis, information on the choice of priors and Markov chain Monte Carlo settings
☒	☐	For hierarchical and complex designs, identification of the appropriate level for tests and full reporting of outcomes
☒	☐	Estimates of effect sizes (e.g. Cohen's *d*, Pearson's *r*), indicating how they were calculated

Our web collection on statistics for biologists contains articles on many of the points above.

Software and code

Policy information about availability of computer code

Data collection	All data are publicly available.
Data analysis	All data analyses were performed using publicly available software.

For manuscripts utilizing custom algorithms or software that are central to the research but not yet described in published literature, software must be made available to editors and reviewers. We strongly encourage code deposition in a community repository (e.g. GitHub). See the Nature Portfolio guidelines for submitting code & software for further information.

Data

Policy information about availability of data

All manuscripts must include a data availability statement. This statement should provide the following information, where applicable:

- Accession codes, unique identifiers, or web links for publicly available datasets
- A description of any restrictions on data availability
- For clinical datasets or third party data, please ensure that the statement adheres to our policy

Data deposition: Raw sequencing reads are available in the NCBI Sequence Read Archive under the BioProject numbers listed in Supplementary Data S1, along with associated specimen information, tissue numbers, and sample locations.
A FigShare Repository contains alignments, trees and code used for data analysis

https://doi.org/10.6084/m9.figshare.25924390.

Research involving human participants, their data, or biological material

Policy information about studies with human participants or human data. See also policy information about sex, gender (identity/presentation), and sexual orientation and race, ethnicity and racism.

Reporting on sex and gender	NA
Reporting on race, ethnicity, or other socially relevant groupings	NA
Population characteristics	NA
Recruitment	NA
Ethics oversight	NA

Note that full information on the approval of the study protocol must also be provided in the manuscript.

Field-specific reporting

Please select the one below that is the best fit for your research. If you are not sure, read the appropriate sections before making your selection.

☐ Life sciences ☐ Behavioural & social sciences ☒ Ecological, evolutionary & environmental sciences

For a reference copy of the document with all sections, see nature.com/documents/nr-reporting-summary-flat.pdf

Ecological, evolutionary & environmental sciences study design

All studies must disclose on these points even when the disclosure is negative.

Study description	This study utilized existing ethanol-preserved tissues from museum collections for DNA extraction.
Research sample	119 species
Sampling strategy	This study utilized existing ethanol-preserved tissues from museum collections for DNA extraction.
Data collection	This study utilized existing ethanol-preserved tissues from museum collections for DNA extraction.
Timing and spatial scale	We used samples from Australia-New Guinea, Indo Pacific, Madagascar, and the New World
Data exclusions	NA
Reproducibility	All analyses are reproducible using the raw sequence data and following described methods
Randomization	NA
Blinding	NA

Did the study involve field work? ☒ Yes ☐ No

Field work, collection and transport

Field conditions	A subadult lesser salmon catfish (Neoarius graeffei) was collected using line fished, 2.5m depth, 22`C
Location	Barratta Creek, Australia
Access & import/export	Frozen/ Formalin-ethanol
Disturbance	NA

Reporting for specific materials, systems and methods

We require information from authors about some types of materials, experimental systems and methods used in many studies. Here, indicate whether each material, system or method listed is relevant to your study. If you are not sure if a list item applies to your research, read the appropriate section before selecting a response.

Materials & experimental systems

n/a	Involved in the study
☒	☐ Antibodies
☒	☐ Eukaryotic cell lines
☒	☐ Palaeontology and archaeology
☐	☒ Animals and other organisms
☒	☐ Clinical data
☒	☐ Dual use research of concern
☒	☐ Plants

Methods

n/a	Involved in the study
☒	☐ ChIP-seq
☒	☐ Flow cytometry
☒	☐ MRI-based neuroimaging

Animals and other research organisms

Policy information about studies involving animals; ARRIVE guidelines recommended for reporting animal research, and Sex and Gender in Research

Laboratory animals	NA
Wild animals	Sea catfish
Reporting on sex	NA
Field-collected samples	A subadult lesser salmon catfish (Neoarius graeffei) was collected from Barratta Creek, Australia (-19.568° S, 147.207° E) on August 7, 2020. Flash-frozen samples (blood, skin, fin clips, gill rakers, muscle) preserved in ethanol and RNA later were sent to the Vertebrate Genome Laboratory (VGL) for DNA/RNA extraction, sequencing, and assembly
Ethics oversight	The University of Oklahoma Institutional Animal Care and Use Committee reviewed and approved protocol #2022-0239, entitled "Investigating the factors shaping marine-derived freshwater fish radiations in tropical rivers of Australia and New Guinea."

Note that full information on the approval of the study protocol must also be provided in the manuscript.

Plants

Seed stocks	NA
Novel plant genotypes	NA
Authentication	NA
