# Supplementary material for: Ecological diversification of sea catfishes is accompanied by genome-wide signatures of positive selection
Source: Nat Commun. 2024 Nov 20;15:10040. doi: 10.1038/s41467-024-54184-3 (PMC11579386; doi:10.1038/s41467-024-54184-3)
Supplement: Supplementary file 3 — Description of Additional Supplementary Files [file 41467_2024_54184_MOESM3_ESM.pdf]

Description of Additional Supplementary Files

Supplementary Data 1

Description: Samples used in the current study, including associated specimen, tissue number, and sample location.

Supplementary Data 2

Description: Information of the 4 subsets used in this study. Genes are specified.

Supplementary Data 3

Description: Habitat occupancy dataset.

Supplementary Data 4

Description: Summary of the genome statistics for the SPAdes preassemblies.

Supplementary Data 5

Description: Results of Benchmarking genome quality using the Universal Single-Copy Orthologs (BUSCO) for the eukaryota_odb10 and actinopterygii_odb10 datasets. Percentage of complete (C), fragmented (F), and missing (M) eukaryota_odb10 and actinopterygii_odb10 single copy orthologs are detailed.

Supplementary Data 6

Description: Summary of the genome statistics for the RagTag assemblies.

Supplementary Data 7

Description: OrthoFinder Statistics.

Supplementary Data 8

Description: Age estimates for MCMCTrees.

Supplementary Data 9

Description: Time spent by ariid lineages in each state on the tree. raw: raw time; prop: proportional time.

Supplementary Data 10

Description: Candidate genes used in this study.

Supplementary Data 11

Description: Colored cells indicate genes under positive selection for the marine to freshwater transitions, considering topological uncertainty, and detected in at least seven out of ten trees. Genes in bold represent PSGs detected in at least nine out of ten trees. GUIS: genes under intensification of selection; GURS: genes under

relaxation of selection.

Supplementary Data 12

Description: Genes under positive selection for the stenohaline to euryhaline transitions, considering topological uncertainty, and detected in at least seven out of ten trees. Genes in bold represent PSGs detected in at least nine out of ten trees. GUIS: genes under intensification of selection; GURS: genes under relaxation of selection.

Supplementary Data 13

Description: Genes under positive selection for the benthic to pelagic planktivore (PP) transitions, considering topological uncertainty, and detected in at least seven out of ten trees. Genes in bold represent PSGs detected in at least nine out of ten trees. GUIS: genes under intensification of selection; GURS: genes under relaxation of selection.

Supplementary Data 14

Description: Comparison of the number of genes under positive selection for the three habitat transitions, analyzed using three different HyPhy methods. Genes were considered under selection if detected in the master tree, at least seven out of ten trees, or nine out of ten trees. GUIS: genes under intensification of selection; GURS: genes under relaxation of selection.

Supplementary Data 15

Description: Number of genes under positive selection for habitat transitions, both during the transition and during and after the transition, along with a comparison between the adaptive radiation and the remaining test clades, considering topological uncertainty. Results are presented for analyses conducted using only the master tree, 9-10 trees, and 7 or more trees.

Supplementary Data 16

Description: Convergent and parallel positively selected sites identified using MEME. Amino acid sites exhibiting convergent or parallel evolution under positive selection, as identified by the mixed effects model of evolution (MEME) in HyPhy (using a likelihood ratio test with a significance threshold of $p < 0.1$).

Supplementary Data 17a

Description: phylANOVA results of the repeat content characterization. Significant p values are highlighted in bold.

Supplementary Data 17b

Description: FDR-corrected p values for phylANOVA results of repeat content characterization. No significant p values were observed prior to correction.
